# Genome-Wide Assessment of Stress-Associated Genes in Bifidobacteria

**DOI:** 10.1128/aem.02251-21

**Published:** 2022-03-21

**Authors:** Marie Schöpping, Tammi Vesth, Kristian Jensen, Carl Johan Franzén, Ahmad A. Zeidan

**Affiliations:** a Systems Biology, Discovery, R&D, Chr. Hansen A/S, Hørsholm, Denmark; b Division of Industrial Biotechnology, Department of Biology and Biological Engineering, Chalmers University of Technology, Gothenburg, Sweden; Washington University in St. Louis

**Keywords:** bifidobacteria, genomics, stress response

## Abstract

Over the last decade, the genomes of several *Bifidobacterium* strains have been sequenced, delivering valuable insights into their genetic makeup. However, bifidobacterial genomes have not yet been systematically mined for genes associated with stress response functions and their regulation. In this work, a list of 76 genes related to stress response in bifidobacteria was compiled from previous studies. The prevalence of the genes was evaluated among the genome sequences of 171 *Bifidobacterium* strains. Although genes of the protein quality control and DNA repair systems appeared to be highly conserved, genome-wide *in silico* screening for consensus sequences of putative regulators suggested that the regulation of these systems differs among phylogenetic groups. Homologs of multiple oxidative stress-associated genes are shared across species, albeit at low sequence similarity. Bee isolates were confirmed to harbor unique genetic features linked to oxygen tolerance. Moreover, most studied Bifidobacterium adolescentis and all Bifidobacterium angulatum strains lacked a set of reactive oxygen species-detoxifying enzymes, which might explain their high sensitivity to oxygen. Furthermore, the presence of some putative transcriptional regulators of stress responses was found to vary across species and strains, indicating that different regulation strategies of stress-associated gene transcription contribute to the diverse stress tolerance. The presented stress response gene profiles of *Bifidobacterium* strains provide a valuable knowledge base for guiding future studies by enabling hypothesis generation and the identification of key genes for further analyses.

**IMPORTANCE** Bifidobacteria are Gram-positive bacteria that naturally inhabit diverse ecological niches, including the gastrointestinal tract of humans and animals. Strains of the genus *Bifidobacterium* are widely used as probiotics, since they have been associated with health benefits. In the course of their production and administration, probiotic bifidobacteria are exposed to several stressors that can challenge their survival. The stress tolerance of probiotic bifidobacteria is, therefore, an important selection criterion for their commercial application, since strains must maintain their viability to exert their beneficial health effects. As the ability to cope with stressors varies among *Bifidobacterium* strains, comprehensive understanding of the underlying stress physiology is required for enabling knowledge-driven strain selection and optimization of industrial-scale production processes.

## INTRODUCTION

Bifidobacteria are high-G+C Gram-positive bacteria that are of industrial importance due to their probiotic effects. When used as probiotics, bifidobacteria are exposed to various types of environmental stressors during their production, storage, and administration, such as oxygen (O_2_), acids, and bile salts (1). To exert their beneficial effects, probiotic strains must retain their viability despite being subjected to these stressors. This makes resistance to environmental stressors an important criterion for the selection of probiotic bifidobacteria for industrial applications.

Over the past 2 decades, the morphological, physiological, and metabolic stress responses of bifidobacteria have been increasingly studied to understand the molecular mechanisms that underlie their stability and robustness (2–32). The stress responses of *Bifidobacterium* strains with diverse stability properties have been assessed, applying traditional methodologies (2–7, 14, 25, 27–32) and, more recently, omics technologies (8–13, 15–24, 26) to characterize and compare strains. The response and tolerance of bifidobacteria to a particular stressor was found to differ significantly among species but also among strains (33, 34). This diversity hampers the extrapolation of data from well-studied strains, such as B. longum subsp. *longum* NCC2705 and B. breve UCC2003, to even closely related strains. However, understanding the molecular mechanisms underlying stability and robustness in bifidobacteria is crucial for driving rational strain selection as well as knowledge-driven optimization of production processes.

Variation in stress tolerance across *Bifidobacterium* strains might be explained by their genetic diversity in terms of stress-associated genes. In 2002, Bifidobacterium longum subsp. *longum* NCC2705 was the first *Bifidobacterium* strain to have its genome sequenced (35). In the following years, multiple genomes of *Bifidobacterium* strains have been sequenced, resulting in more than 150 complete genome sequences and even more draft sequences being publicly available. Previous comparative analyses of bifidobacterial genomes have mainly focused on the evolution of the genome and niche-specific adaptations, paying special attention to their carbohydrate utilization capabilities (36–44). Some genomic studies have focused on the entire genus (36–42), whereas others have targeted individual species (43, 44). A number of studies highlighted the high genomic diversity across *Bifidobacterium* species (38, 39), but to date only a single study has touched upon the prevalence of stress-associated genes in bifidobacteria (42).

The aim of the present study was to explore the prevalence of known stress-associated genes among bifidobacteria with publicly available genome sequences to gain insights into the diversity of stress physiology in this important group of bacteria. The obtained stress response gene profiles were combined with the current knowledge on stress tolerance of *Bifidobacterium* strains to identify genotype-phenotype correlations. In addition, previous findings on the stress response mechanisms were assessed, in terms of their genus-wide validity, and novel hypotheses were generated.

## RESULTS AND DISCUSSION

In this study, the prevalence of stress-associated genes in bifidobacterial genomes was investigated to gain new insight into the diverse stress physiology of the genus. A list of 76 genes that have been previously implicated in stress responses of *Bifidobacterium* strains was compiled through an extensive literature survey. The genes were grouped into six categories: (i) protein quality control (PQC) and DNA repair systems, (ii) oxidative stress, (iii) acid stress, (iv) bile stress, (v) organic solvent stress, and (vi) putative transcriptional regulators of stress response. With the single exception of the glutamate/gamma-aminobutyrate antiporter GadC, genes encoding transporters were not included in the study due to the difficulty of computationally determining the substrate specificity based solely on their protein sequence. All stress-associated genes included in this study are listed in Table S1 in the supplemental material.

The distribution of the selected genes was assessed in 171 *Bifidobacterium* strains (Table S2), representing 22 species (Table 1), through protein homology search. In total, 9,362 hits were identified. The complete list of homologs is provided in Data Set S1.

**TABLE 1:** *Bifidobacterium* species included in the study^*a*^

Species	No. of strains	Prevailing isolation source
Bifidobacterium actinocoloniiforme	1	Digestive tract content of *Bombus lucorum* (bumblebee)
Bifidobacterium adolescentis	8	Feces of human adults; bovine rumen; sewage
Bifidobacterium angulatum	2	Sewage; feces of human adults
Bifidobacterium animalis (not assigned to subspecies)	4	
Bifidobacterium animalis subsp. *animalis*	3	Feces of rats and guinea pigs
Bifidobacterium animalis subsp. *lactis*	21	Feces of chickens and rabbits; fermented milk; sewage
Bifidobacterium asteroides	2	Intestine of Apis mellifera subsp. *caucasica*, *ligustica*, and *mellifera* (honeybee)
Bifidobacterium bifidum	10	Feces of human adults and infants and suckling calves; human vagina
Bifidobacterium breve	46	Feces of infants and suckling calves
Bifidobacterium catenulatum (not assigned to subspecies)	2	
Bifidobacterium catenulatum subsp. *kashiwanohense*	2	Feces of infants and human adults; human vagina; sewage
Bifidobacterium choerinum	1	Feces of piglets; sewage
Bifidobacterium coryneforme	1	Intestine of Apis mellifera subsp. *mellifera* (honeybee)
Bifidobacterium dentium	3	Human dental caries and oral cavity; feces of human adults; human vagina
Bifidobacterium eulemuris	1	Feces of *Eulemuris macaco* (black lemur)
Bifidobacterium indicum	1	Intestine of Apis cerana (honeybee)
Bifidobacterium kashiwanohense	1	Feces of a healthy infant (1.5 yr old)
Bifidobacterium lemurum	1	Feces of *Lemur catta* (ring-tailed lemur)
Bifidobacterium longum (not assigned to subspecies)	12	
Bifidobacterium longum subsp. *infantis*	11	Feces of infants and suckling calves; human vagina
Bifidobacterium longum subsp. *longum*	27	Feces of human adults and infants and suckling calves; human vagina; sewage
Bifidobacterium longum subsp. *suillum*	1	Feces of piglets
Bifidobacterium pseudocatenulatum	2	Feces of infants and suckling calves; sewage
Bifidobacterium pseudolongum (not assigned to subspecies)	2	
Bifidobacterium pseudolongum subsp. *globusum*	1	Feces of lambs, piglets, rabbits, rats, and suckling calves; bovine rumen; sewage
Bifidobacterium pullorum subsp. *gallinarum*	1	Feces of canine
Bifidobacterium scardovii	1	Human blood
Bifidobacterium subtile	1	Sewage; human carious lesions
Bifidobacterium thermophilum	2	Feces of chickens, pigs, and suckling calves; bovine rumen; sewage

a *Bifidobacterium* strains of 22 species were included in the study. All *Bifidobacterium* strains included in the study can be found in Table S2. The prevailing isolation source has been adapted from Mattarelli and Biavati (61).

**FIG 1:**
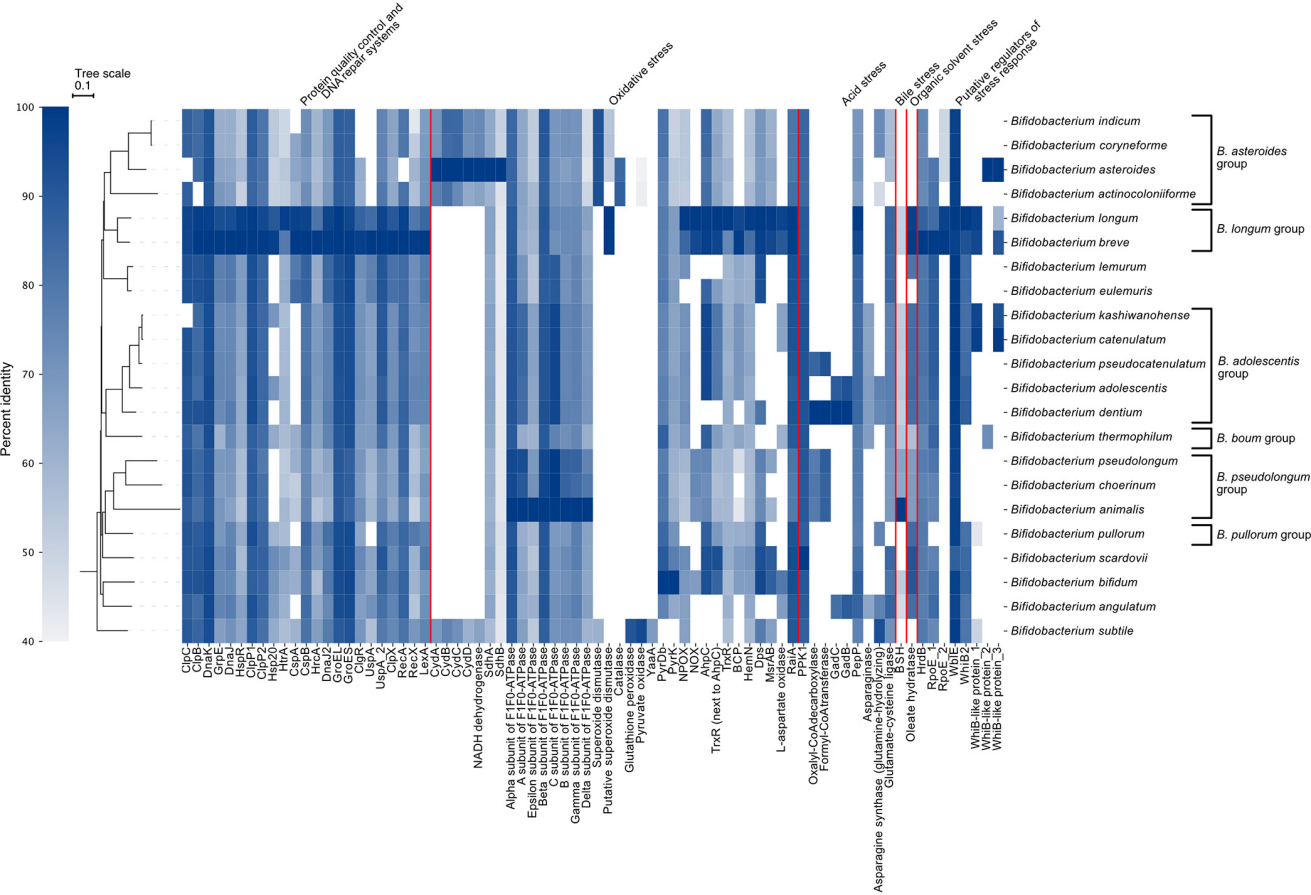
Heat map representing the median sequence identity of the best hit of 76 stress-associated gene products in 22 *Bifidobacterium* species, including 171 *Bifidobacterium* strains. The analyzed *Bifidobacterium* species are members of six previously suggested phylogenetic groups (37, 40). For each stress-associated gene, a query protein sequence was extracted from the genome of a strain in which it was proposed to be involved in stress responses. Homologs of stress-associated gene products across the 22 species were identified using DIAMOND BLASTp (E value, 0.001; sequence identity cutoff, 40%; coverage cutoff, 70%). The maximum likelihood phylogeny tree was constructed using CLC Genomics Workbench. The tree scale gives the average number of substitutions per site. Information on the stress-associated gene products can be found in Table S1.

**FIG 2:**
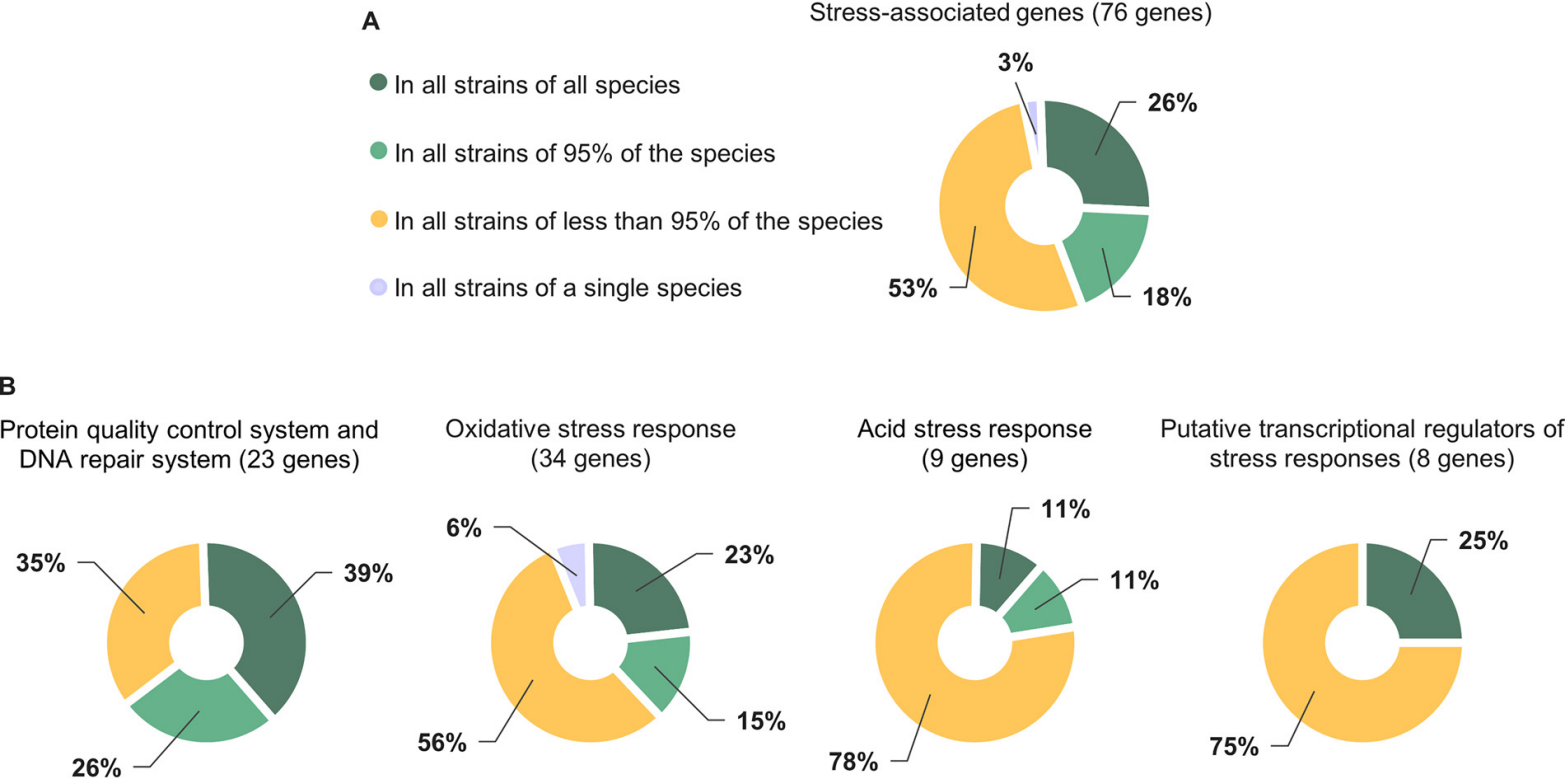
Distribution of the stress-associated genes in the analyzed *Bifidobacterium* species. Overall, 171 *Bifidobacterium* strains were studied, representing 22 species. (A) Proportion of genes found in all strains of all species, in all strains of 95% of the species (21 out of 22 species), in all strains of less than 95% of the species, and in all strains of a single species. (B) Proportion of genes found in all strains of all species, in all strains of 95% of the species, in all strains of less than 95% of the species, and in all strains of a single species in the categories (i) protein quality and DNA repair systems, (ii) oxidative stress response, (iii) acid stress response, and (iv) putative transcriptional regulators of stress responses. The single genes analyzed in the categories organic solvent and bile stress are present in strains of less than 21 species. All species-specific genes were identified in *B. subtile* KCTC 3272, using genes from *B. tibiigranuli* TMW 2057 as query genes.

Clear differences in the presence of stress-associated genes were detected among previously suggested phylogenetic groups of bifidobacteria (37, 40) and among species (Fig. 1). In addition, a few differences were observed among strains of the same species (Fig. S1). Slightly fewer than half of the studied stress-associated genes were found in all studied strains or missing in strains of only 1 out of the 22 species (i.e., present in 95% of the species), whereas 53% of the studied stress-associated genes were identified in fewer than 95% of the species (Fig. 2A). Homologs of two studied genes (3%) were only identified in *B. subtile* KCTC 3272 (Fig. 2A), which was the only representative of the *B. subtile* species. The analysis showed that most genes associated with the PQC and DNA repair systems, i.e., 64%, are well conserved across species, whereas more than half of the genes related to oxidative stress, acid stress, and putative transcriptional regulators of stress responses, i.e., 62%, 78%, and 75%, respectively, were present in fewer than 95% of species (Fig. 2B). The degree of sequence similarity of the stress-associated genes across species was highly diverse (Fig. 1). Cutoffs of 40% sequence identity and 70% coverage were applied for the homology search to reduce the risk of including homologs with function dissimilar to that of the query protein. Most of the identified homologs shared at least 50% sequence identity (Fig. 1), and the alignment covered more than 80% of the query stress protein sequence (Data Set S1). However, for some stress-associated genes the sequence similarity was lower across some homologs, particularly for query protein sequences or hits from the genomes of strains that belongs to the *B. asteroides* group.

To investigate the correlation between the presence of stress-associated genes and actual stress tolerance of *Bifidobacterium* strains, data on the tolerance to individual stressors was collected (see Table S3). In the following sections, each category of stress-associated genes will be discussed along with the current knowledge on the studied genes and the homologs identified in this study. When very low protein sequence similarity was detected across homologs, the sequences of the homologs were more thoroughly compared through multiple-sequence alignment.

### Protein quality control and DNA repair systems.

Different stresses, such as oxidative and heat stress, have a detrimental effect on macromolecules, including DNA and proteins, making the PQC system and DNA repair systems crucial for a cell’s stress resistance (45, 46). So-called heat shock proteins (Hsps), which function as chaperones, cochaperones, and proteases, are major constituents of the PQC system in bacteria (46). Despite their naming, they are also involved in responses to stresses other than heat (46).

### (i) Most genes of the protein quality control and DNA repair systems are highly conserved.

Most information available on the genomic organization and regulation of Hsps in bifidobacteria derives from extensive studies on B. breve UCC2003 exposed to heat, osmotic, and organic solvent stress (24, 47). Based on genetic and transcriptional studies, a model for the stress gene regulatory network, covering the regulation of the PQC system, has been proposed (24). The model includes 11 Hsps (ClpC, ClpP1, ClpP2, GroEL, GroES, ClpB, ClpX, DnaK, GrpE, DnaJ, and DnaJ2) and three transcriptional regulators (the repressors HspR and HrcA and the activator ClgR) (24). The transcriptional regulation of Hsps has been proposed to overlap those of the DNA repair system (24). Based on the high degree of conservation of the regulons and binding sites, the model of the stress gene regulatory network in B. breve UCC2003 was suggested to be valid for all *Bifidobacterium* species characterized (24). Moreover, the presence of cold shock protein A (CspA), encoded next to GroEL (31), and universal stress protein A (UspA), encoded next to ClpC in B. breve UCC2003, was suggested to be widely conserved in bifidobacteria (6). Since the availability of bifidobacterial genomes was still rather limited at the time of these studies, we assessed the general validity of the proposed model using the genome sequences available today.

Searching all 171 bifidobacterial genomes for orthologs of the genes encoding the described PQC and DNA repair systems in B. breve UCC2003 confirmed that, with very few exceptions, all analyzed *Bifidobacterium* strains possess all these genes (Fig. 1 and Fig. S1). However, the degree of sequence conservation varied widely with the different genes (Fig. 1), suggesting that the regulatory network is of fundamental importance in bifidobacteria but might have some unknown functional variations across species.

In contrast to previous findings that the *cspA-groEL* organization and *uspA-clpC* organization are widely conserved in bifidobacteria (6, 31), our genomic analysis revealed that CspA and UspA were present in less than 95% of the bifidobacterial genomes. A homolog of UspA was not detected in strains isolated from the digestive tract of bees (*B. asteroides* group) in *B. pullorum* subsp. *gallinarum* CACC 514 (only representative of *B. pullorum* species) (Fig. 1) or in some strains of B. breve (Fig. S1). Moreover, all strains belonging to the B. adolescentis group, *B. angulatum*, *B. indicum* LMG 11587 (only representative of *B. indicum* species), and *B. pullorum* subsp. *gallinarum* CACC 514 (Fig. 1), and some strains of B. breve lacked a homolog of CspA (Fig. S1). The lack of sufficient knowledge on the function of CspA and UspA in bifidobacteria makes the biological implication of the absence of these genes ambiguous. In general, cold shock proteins might serve as RNA chaperones under stress conditions (48), and universal stress proteins might have a role in DNA protection (49). In B. breve UCC2003, the *cspA* gene was induced upon heat stress (24, 31), whereas the *uspA* gene was not (6). Interestingly, a second universal stress protein gene (UspA_2), upregulated upon bile stress in B. breve UCC2003 (19), and a gene encoding a cold shock domain-containing protein (CspB), located on the gene segment between *groEL* and *clpC*, were found to be conserved in all strains of all studied species (Fig. 1). Although the role of CspB remains to be elucidated, it seems not to be involved in the heat stress response in bifidobacteria (50).

Some proposed characteristics of the bifidobacterial heat shock system are uncommon for high-G+C Gram-positive bacteria (6, 31). High-G+C Gram-positive bacteria, such as members of the genus *Streptomyces*, contain several copies of *clpC* (51) and *groEL* (52), whereas bifidobacterial genomes were suggested to contain only a single copy of these genes (6, 31). This was confirmed for all genomes included in this study. Furthermore, additional members of the Hsp100 family, including ClpA, ClpE, and ClpY as well as heat shock proteins of the Hsp33 and Hsp90 families, were confirmed to be absent from all sequenced bifidobacterial genomes based on functional annotation information in the NCBI Reference Sequence (RefSeq) database (47).

### (ii) The regulation of the protein quality control and DNA repair systems may vary across phylogenetic groups.

The regulatory network of the PQC and DNA repair systems in B. breve UCC2003 was suggested to be under the control of the transcriptional repressors HspR (heat shock protein repressor) and HrcA (heat regulation at controlling inverted repeat of chaperone expression [CIRCE]) as well as the transcriptional activator ClgR (Clp gene regulator) (24). The proposed consensus motifs of the regulators and their position in the genome of B. breve UCC2003 are shown in Table 2. The model proposed for the regulation of the PQC and DNA repair systems in bifidobacteria is highly based on these binding motifs and their position in the genome of B. breve UCC2003. Thus, after confirming the high degree of conservation of the included genes, the general validity of the proposed model was further reviewed by assessing the conservation of the binding motifs of the regulators across different strains of the genus. For this purpose, the genome sequences B. longum subsp. *longum* NCC2705, belonging to the same phylogenetic group as B. breve UCC2003, and *B. animalis* subsp. *lactis* BB-12 and B. adolescentis ATCC 15703, representing two other phylogenetic groups (Fig. 1) with different stability characteristics (Table S3), were screened for the three motifs using Find Individual Motif Occurrences (FIMO) (53).

**TABLE 2:** Candidate binding sites of the transcriptional regulators HspR, HrcA, and ClgR in the genomes of *Bifidobacterium* strains that represent different phylogenetic groups

Binding site	B. breve UCC2003^*d*^	B. longum NCC2705^*e*^	*B. animalis* BB-12^*e*^	B. adolescentis ATCC 15703^*e*^
HspR (HAIR consensus sequence: AAAsTTGAGysw-N_6_-CTCAAsTTTT^*a*^^,^^*b*^)	DnaK, ×2	DnaK, ×2, (+/−)	DnaK, (−/+)	DnaK, ×2, (−/+), (−)
	ClgR	ClgR, (−)	×	×
	ClpB	ClpB, (−/+)	ClpB, (−/+)	ClpB, (−/+)
HrcA (CIRCE consensus sequence: TTAGCACTC-N_9_-GAGTGCTAA^*a*^^,^^*c*^)	GroEL	GroEL, (+/−)	GroEL, (+/−)	HrcA, (+/−)
	GroES, ×2	GroES, ×2 (+/−)	GroES, (+/−)	GroES, ×2, (+/−)
	HrcA	HrcA, (+/−)	HrcA, (+/−)	HrcA, (+/−)
ClgR (consensus sequence: TNCGCT-N_3_-GGCGNAA^*a*^)	ClpP1	ClpP1, (−)	×	ClpP1, (−)
	HrcA	HrcA, (−)	×	×
	ClpC	ClpC, (+)	×	ClpC, (+)

a Consensus sequences of the motifs have been previously proposed by Zomer et al. (24). The nucleotide codes of the motifs follow the IUPAC nomenclature.

b HAIR, HspR-associated inverted repeats. The number of Ns in the HAIR sequence was incorrectly given as five in the original publication.

c CIRCE, controlling inverted repeat of chaperone expression sequence.

d The binding sites in the genome of B. breve UCC2003 have been determined by Zomer et al. by applying comparative sequence analysis and electrophoresis mobility shift assays (24).

e Candidate binding sites in the genomes of B. longum subsp. *longum* NCC2705, *B. animalis* subsp. *lactis* BB-12, and B. adolescentis ATCC 15703 were determined using the online version of Find Individual Motif Occurrences. ×2, motif has been detected twice in the promoter region of the gene; ×, no motif detected in the promoter region of the gene; +, motif on the plus strand of the genome; −, motif on the minus strand of the genome.

The gene organization of the PQC and DNA repair systems was confirmed to be identical to that in B. breve UCC2003 in all three strains, except for the absence of a CspA homolog in B. adolescentis ATCC 15703. As in B. breve UCC2003, an HspR-associated inverted repeat (HAIR) motif was detected twice upstream of the *dnaK* operon in B. longum subsp. *longum* NCC2705 and B. adolescentis ATCC 15703 (Table 2). In contrast, in *B. animalis* subsp. *lactis* BB-12, the motif appears only once in the promoter region of *dnaK* and shows slight deviations from the suggested consensus sequence (ATACTTGAGTGA-N_6_-CTCAAGTTTT), with a tyrosine inserted into the adenine-rich extension. The presence of a single HAIR motif upstream of the *dnaK* operon was described for *B. animalis* subsp. *lactis* DSM 10140 (4), suggesting that this is a species-wide characteristic. No HAIR motif could be detected upstream of the *clgR* gene in *B. animalis* subsp. *lactis* BB-12 and B. adolescentis ATCC 15703 (Table 2), whereas in B. longum subsp. *longum* NCC2705 the HAIR motif upstream of the *clgR* gene lies inside the coding region of a gene predicted to encode a CinA family protein. Genome-wide *in silico* screening for the motif further revealed that, unlike the other strains, *B. animalis* subsp. *lactis* BB-12 only has one CIRCE-like motif upstream of the *groES* gene (Table 2). Several nonsignificant hits for the ClgR-like motif were detected in B. longum subsp. *longum* NCC2705, *B. animalis* subsp. *lactis* BB-12, and B. adolescentis ATCC 15703. Therefore, the sequences upstream of the potentially ClgR-regulated genes were screened for the ClgR motif in individual analyses. A ClgR-like motif was detected in the promoter region of the *clpP* operon, upstream of *hrcA* and *clpC* in B. longum subsp. *longum* NCC2705 (hit upstream of HrcA has a q-value above 0.01), but not in *B. animalis* subsp. *lactis* BB-12. In B. adolescentis ATCC 15703, the ClgR motif was only detected upstream of the *clpC* gene and the *clpP* operon (Table 2).

Taken together, these findings suggest that the regulation of the PQC and DNA repair systems in *B. animalis* subsp. *lactis* BB-12 and B. adolescentis ATCC 15703 differs from the regulation in B. breve UCC2003 and B. longum subsp. *longum* NCC2705. In particular, the function of the activator ClgR in *B. animalis* subsp. *lactis* BB-12 and B. adolescentis remains obscure. Consequently, the proposed model of the stress regulatory network might be valid for rather closely related strains of B. breve UCC2003 but not for all strains of the genus. Thus, the regulation of the PQC and DNA repair systems merits further research, with a focus on strains outside the B. longum group.

### (iii) Multiple species lack an Hsp20 homolog.

Besides the already-described genes, the serine protease HtrA and the small heat shock protein Hsp20 were suggested to be part of the stress-induced PQC system. Both bile and severe heat stress were observed to induce the expression of *htrA* in *Bifidobacterium* strains (21, 22, 50, 54). Homologs of HtrA were found to be present in all analyzed genomes except three B. longum strains (Fig. S1). Hsp20 was found to be strongly induced upon multiple stresses, including heat, high osmolality, hydrogen peroxide (H_2_O_2_), and starvation, and was proposed as a suitable biomarker for stress (13, 30). The analysis of this study showed that only 73% of the *Bifidobacterium* strains possess genes encoding homologs of Hsp20 from B. breve UCC2003. The presence of an Hsp20 homolog seems to be species dependent, except for that in B. longum strains (Fig. S1). In contrast to the previous hypothesis that Hsp20 is exclusive for isolates from the human intestine (30), an Hsp20 homolog was also identified in isolates from bees, such as *B. asteroides* PRL2011 (51% identity, 99% coverage) (Fig. 1 and Data Set S1). However, no Hsp20 homolog was found in other animal isolates (Fig. 1), including *B. animalis* subsp. *lactis* BB-12. Furthermore, the analysis contradicts previous results of a slot plot hybridization test, demonstrating the presence of an *hsp20* gene in the human isolate B. catenulatum DSM 16992 (LMG 11043) (30); no gene encoding Hsp20 was identified in any B. catenulatum strain, including DSM 16992 (Fig. 1). Since animal isolates, including *B. animalis* subsp. *lactis* BB-12, possess high heat tolerance (33), it can be excluded that Hsp20 is essential for sufficient protection of the cells against heat shock. Rather, Hsp20 may be important for coping with stressors other than heat, as suggested before (30). In other organisms, small heat shock proteins, such as Hsp20, have been linked to functions other than classical chaperones, e.g., biofilm formation and cell protection during dormancy (55). Interestingly, animal isolates have been shown to commonly grow at higher temperatures than human isolates (up to 45°C) (56). Therefore, it is conceivable that high expression of Hsp20 upon heat stress is linked to a slow down of growth in bifidobacteria, as has been observed for HspX in Mycobacterium tuberculosis (57). To increase the understanding of the relationship between the presence of Hsp20 and the stress resistance of *Bifidobacterium* strains, its biological function should be further investigated, e.g., by studying its transcriptional regulation (30) as well as the effect of knocking out the gene on growth and survival at elevated temperatures.

### Oxidative stress response.

As for other stressors, the sensitivity to oxidative stress varies significantly among *Bifidobacterium* strains, but the molecular basis of this variation is not fully understood (33, 58). The toxicity of O_2_ is mainly linked to the formation of so-called reactive oxygen species (ROS), including H_2_O_2_, superoxide anions (O_2_^·−^), and hydroxyl radicals (OH^·^). In contrast to aerobic bacteria, O_2_-sensitive bacteria lack an efficient system to detoxify ROS. For the elimination of oxidative stress, the interplay of O_2_-scavenging and ROS-detoxifying enzymes is critical to avoid accumulation of H_2_O_2_, which is produced by some of these enzymes and decomposed by others.

In line with the different O_2_ tolerances of *Bifidobacterium* strains, most of the 34 investigated genes that are related to oxidative stress were not conserved across species (Fig. 1). In addition, some oxidative stress-associated genes had very low protein sequence similarity across species (Fig. 1), as discussed further below.

### (i) Bee-specific strains harbor unique genetic characteristics linked to oxygen tolerance.

Isolates from the digestive tract of bees have been shown to have exceptionally high O_2_ tolerance (Table S3) (59), which might derive from adaptation to elevated O_2_ concentrations in their natural environment (60). Previous genome analysis of the aerotolerant *B. asteroides* PRL2011 revealed genes encoding enzymes of an electron transport chain for aerobic respiration (59), which had also been detected in additional bee isolates (39). In agreement, we identified homologs for a H^+^-translocating NADH dehydrogenase, cytochrome *d* oxidase (CydA, CydB, CydC, and CydD), succinate dehydrogenase subunits (SdhA and SdhB), and all subunits of F_1_F_0_-ATPase in all bee isolates included in the analysis. Homologs of all these proteins were further found in *B. subtile* KCTC 3272 (Fig. 1), an isolate from sewage, the living host of which remains unknown (61).

Despite the presence of genes encoding an electron transport chain in all four strains, O_2_ consumption was previously only detected in *B. asteroides* PRL2011 but not in *B. coryneforme* LMG 18911, *B. indicum* LMG 11587, and *B. actinocoloniiforme* DSM 22766 (59). The absence of other respiratory-associated genes in these strains, such as the hydrogen peroxide-dependent heme synthase (based on functional annotation information in the NCBI database), may explain the observed phenotypical difference.

Homologs of the succinate dehydrogenase subunits SdhA and SdhB were detected in all studied genomes (Fig. 1), suggesting a housekeeping function (59). As all strains lacked homologs of the heme-binding membrane domain subunit (SdhC and SdhD) of the enzyme, it appears to exist as a soluble enzyme. Low sequence similarity (42 to 67%) was detected between the genes encoding SdhA and SdhB from *B. asteroides* PRL2011 (query) and their homologs in other species (Fig. 1). In nonrespiring strains, the enzyme might rather function as fumarate reductase, catalyzing the opposite reaction of succinate dehydrogenase (62).

The F_1_F_0_-ATPase (ATP synthase) was highly conserved across genomes of the investigated species (Fig. 1). In general, F_1_F_0_-ATPase can catalyze the synthesis of ATP using the energy of an electrochemical ion gradient or convey the extrusion of protons under low driving force conditions, such as acid stress, using ATP for energy supply (63). In nonrespiring strains, the enzyme is thought to be crucial for the maintenance of a proton gradient (64), whereas in respiring strains, such as *B. asteroides* PRL2011, it might contribute to ATP synthesis as part of the electron transport chain (59). The sequences of the alpha- and beta-subunits, forming the catalytic site of the enzyme in the F_1_ domain, were found to be highly conserved across strains in our analysis. In contrast, relatively low sequence similarity was detected for other subunits of the F_1_F_0_-ATPase, particularly for the epsilon subunit (part of the rotor) and the delta subunit (part of the stator) of F_1_F_0_-ATPase (Fig. 1).

Most *Bifidobacterium* strains are superoxide dismutase (SOD) negative and catalase negative. However, genes predicted to encode SOD and catalase have been identified in the bee isolates, e.g., *B. xylocopae* subsp. nov. XV2 and *B. asteroides* PRL2011 (59, 65), and an oxygen-inducible heme catalase has been characterized in *B. asteroides* DSM 20089 (66). Our genomic analysis revealed that all strains that harbor genes for a putative electron transport chain also harbor homologs of SOD from *B. xylocopae* XV2 (Fig. 1), whereas only *B. asteroides* and *B. actinocoloniiforme* DSM 22766 (the only representative of *B. actinocoloniiforme* species) were found to have a catalase homolog (Fig. 1). The SOD and catalase genes in these strains showed high sequence identity (91% at 100% coverage) to SOD genes from the bee isolate Bombiscardovia coagulans (67).

Even though homologs of SOD from *B. xylocopae* XV2 were only detected in bee isolates, previous studies have reported SOD activity, albeit very low, in *Bifidobacterium* strains isolated from other sources (68–70). Spontaneous destruction of O_2_^·−^ might explain low SOD activity in strains lacking an SOD homolog (71). Nonetheless, we searched bifidobacterial genomes in NCBI GenBank for the presence of genes annotated as SOD aside from the SOD homologs from *B. xylocopae* XV2 and detected a gene predicted to encode an SOD in B. longum subsp. *infantis* ATCC 15697 (Blon_1406, WP_003829426.1). This strain has been reported to have low SOD activity and to be hypersensitive to O_2_ (28, 69, 70). The putative SOD gene from B. longum subsp. *infantis* ATCC 15697 is significantly shorter than the SOD homologs in bee isolates (132 versus 206 amino acids). The putative SOD homolog was included in our homology search. Homologs with high sequence similarity to the putative SOD gene were detected in a subset of B. breve strains and B. longum subsp. *infantis* strains as well as in four bee isolates, in the latter with only 50% protein sequence identity (Fig. S1). It remains to be confirmed if the gene actually encodes an SOD that might negatively influence the O_2_ tolerance in strains lacking a sufficient H_2_O_2_ detoxification system.

### (ii) Oxidative stress-associated genes from *B. tibiigranuli* are rare across species.

Genomic analysis of the water kefir isolate *B. tibiigranuli* TMW 2.2057 identified genes, including pyruvate oxidase, glutathione peroxidase, and the peroxide stress protein YaaA, that potentially contribute to O_2_ tolerance (18). While homologs of all three gene products were found in *B. subtile* KCTC 3272, no homologs for glutathione peroxidase and YaaA were found in strains of another species (Fig. 1), and only homologs with very low similarity to pyruvate oxidase (40 to 41%) were identified in some bee isolates (Fig. 1). These results confirm the previous findings that *B. tibiigranuli* strains have several unique genetic traits among bifidobacteria and that the species is phylogenetically close to *B. subtile* (18).

### (iii) The bifidobacterial HemN might function as HemW.

Under aerobic conditions, the expression of a gene predicted to encode an oxygen-independent coproporphyrinogen III oxidase (HemN) was induced in the O_2_-tolerant *B. animalis* subsp. *lactis* IPLA4549 and an *ahpC*-overexpressing mutant of B. longum subsp. *longum* NCC2705 (20, 27). Our genomic analysis detected homologs of HemN from B. longum subsp. *longum* NCC2705 in all analyzed strains, with lowest sequence similarity (55 to 59%) to the reference in members of the B. pseudolongum and *B. asteroides* group (Fig. 1).

The role of HemN (a key enzyme in heme biosynthesis) in the oxidative stress response remains to be understood (20). In contrast to its annotation in the literature, the HemN homolog from multiple strains was annotated as radical SAM family heme chaperone HemW based on the Prokaryotic Genome Annotation Pipeline (PGAP) functional annotation (Table S1). This held true for HemN homologs from B. longum subsp. *longum* NCC2705 as well as from 33 additional B. longum strains, *B. coryneforme* LMG18911, and *B. indicum* LMG 11587 (Data Set S1). In line with this, in Lactococcus lactis, a protein originally annotated as HemN was previously found to lack coproporphyrinogen III oxidase activity and was instead assigned to the protein HemW family with a putative function as a heme chaperone (72).

**FIG 3:**
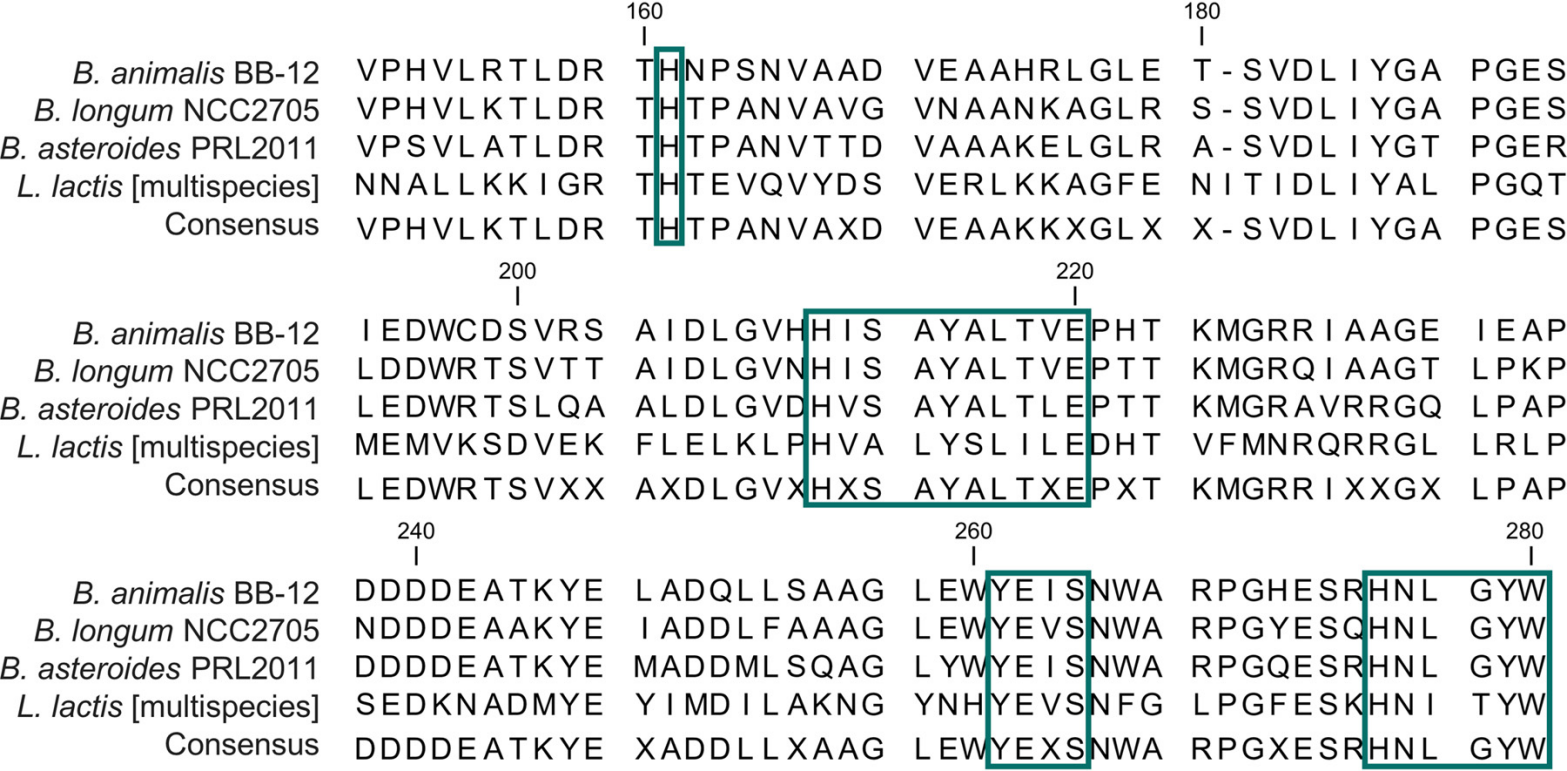
Identification of HemW-specific motifs in HemN homologs of *B. animalis* BB-12, B. longum NCC2705, and *B. asteroides* PRL2011. To assess the presence of HemW-specific motifs in the protein sequences of HemN homologs from *B. animalis* subsp. *lactis* BB-12 (WP_004217870.1), B. longum subsp. *longum* NCC2705 (WP_008783712.1), and *B. asteroides* PRL2011 (WP_033511215.1), their protein sequences were aligned with HemW of L. lactis (WP_003132086.1). The alignment was generated using CLC Genomics Workbench 20.0, default settings, alignment mode set to very accurate. Blue boxes are HemW-specific conserved amino acid residues that distinguish the protein from HemN, identified in L. lactis: H^134^, H^184^xxxYxLxxE, Y^234^ExS, and especially H^248^NxxYW (72).

Due to the inconclusive annotation of the homologs in *Bifidobacterium* strains, its protein sequence in B. longum subsp. *longum* NCC2705, *B. animalis* subsp. *lactis* BB-12, and *B. asteroides* PRL2011 was scanned for conserved amino acid motifs specific to the HemW family to investigate if HemN in bifidobacteria also belongs to the HemW family. All HemW-specific motifs were detected in the gene products of the three strains (Fig. 3), suggesting that also in bifidobacteria, the enzyme does not function as HemN but rather shares its function with HemW in L. lactis.

However, L. lactis strains can respire in the presence of exogenous hemin, whereas most *Bifidobacterium* strains lack enzymes required for respiratory growth, which means that the function of HemW in bifidobacteria appears superfluous. Moreover, coproporphyrinogen III oxidase activity has been detected in cell extract of *B. animalis* subsp. *lactis* IPLA 4549 (20). Assessing the heme-binding affinity of the bifidobacterial HemN/W as well as the effect of heme addition on its expression and on the O_2_ tolerance of strains might help to understand the role of HemN/W in bifidobacteria.

### (iv) H_2_O_2_-forming NAD(P)H oxidases are encoded in both oxygen-tolerant and -sensitive species.

In many studies, low oxidative stress tolerance in bifidobacteria has been linked to H_2_O_2_ formation and its insufficient detoxification under aerobic conditions (69, 70, 73, 74). To date, two H_2_O_2_-forming NAD(P)H oxidases have been characterized in *Bifidobacterium* strains: a *b-*type dihydroorotate dehydrogenase (DHOD) from the O_2_-sensitive B. bifidum ATCC 29521 and an NADPH oxidase (NPOX) from the O_2_-hypersensitive B. longum subsp. *infantis* ATCC 15697 (28, 29). Even though these enzymes have been suggested to contribute to O_2_ sensitivity (28, 29), homologs of DHOD (PyrK and PyrDb subunit) and NPOX were also identified in the genomes of O_2_-tolerant strains, such as *B. animalis* subsp. *lactis* BB-12 (Fig. S1). Overall, 94% and 89% of the analyzed strains possessed PyrK, PyrDb, and NPOX homologs (Fig. S1). Therefore, it can be concluded that the sole presence of these genes is not indicative of O_2_ sensitivity. It seems more likely that the expression level of H_2_O_2_-forming NAD(P)H oxidases (28) and, in their presence, the activity of H_2_O_2_-detoxifying enzymes determine a strain’s ability to cope with O_2_. In line with this, a previous study reported that heterologous expression of *npoxA* from *B. infantis* ATCC 15697 in the O_2_-tolerant strain *B. minimum* DSM 20102 resulted in growth inhibition at O_2_ concentrations above 10% (28). *B. minimum* DSM 20102 has not been included in the homology search of our study due to its incomplete genome sequence at NCBI, but a separate online BLASTp search, using default settings, revealed that *B. minimum* DSM 20102 possesses an NPOX homolog itself, which most likely is not expressed at high levels under aerobic conditions.

The lowest sequence similarity (<60%) was detected for NPOX homologs in strains of the B. pseudolongum and *B. asteroides* group. Multiple-sequence alignment of all identified NPOX hits revealed that the homologs in *B. animalis* possess a unique motif (P-xxx-S/T-xxxxx-C, positions 59 to 71) that is similar to the active-site motif of peroxiredoxins (75). Thus, the activity of the NPOX homologs in these species might vary from that in other species.

Only B. longum subsp. *longum* NCC2705, described to possess relatively high intrinsic H_2_O_2_ tolerance (58), and a few other B. longum and B. bifidum strains were found to lack an NPOX homolog (Fig. S1). It is possible that the absence of the H_2_O_2_-forming activity of NPOX reduces the burden on the strain’s H_2_O_2_-detoxifying enzymes from intracellularly formed H_2_O_2_ and free capacity for the detoxification of extracellular H_2_O_2_. Therefore, it should be assessed if the strains lacking a homolog of NPOX show higher O_2_ tolerance than other strains of the species.

Another enzyme that might contribute to H_2_O_2_ formation is the l-aspartate oxidase, which catalyzes the first step of *de novo* NAD biosynthesis by oxidizing aspartate to iminoaspartate. It has been suggested that both fumarate and O_2_ can serve as electron acceptors for l-aspartate oxidase, while using the latter results in the formation of H_2_O_2_ (76). Even though a previous study showed that fumarate is the preferred *in vivo* substrate of l-aspartate oxidase in Escherichia coli, H_2_O_2_ formation was detected under aerobic conditions when fumarate levels were low (76). Thus, the activity of l-aspartate oxidase might contribute to O_2_ sensitivity by facilitating H_2_O_2_ formation. In line with this conclusion, our results indicated that homologs of l-aspartate oxidase are present primarily in species known to be rather O_2_-sensitive, whereas relatively O_2_-tolerant species, such as *B. animalis*, lacked an l-aspartate oxidase homolog (Fig. 1).

### (v) The absence of multiple ROS detoxification enzymes may cause hypersensitivity to oxygen.

Until now, no H_2_O-forming NADH oxidase (NOX) has been purified and characterized from a *Bifidobacterium* strain. However, an NADH oxidase upregulated in the O_2_-tolerant *B. animalis* subsp. *lactis* IPLA4549 upon oxidative stress was suggested to contribute to detoxification of O_2_ to H_2_O (20). Since the genome sequence of *B. animalis* subsp. *lactis* IPLA4549 is not publicly available, NOX from B. longum subsp. *longum* NCC2705 was used as a query gene. Based on our genomic analysis, all strains of 73% of the studied species lacked a homolog of the putative H_2_O-forming NADH oxidase (Fig. 1). A previous study on the H_2_O-NADH oxidase of Enterococcus faecalis 10C1 revealed that a single cysteine (Cys42) in the active site is determinative of the four-electron reduction of O_2_ to H_2_O in the NADH oxidase (77). Multiple-sequence alignment of the bifidobacterial NOX and the NOX of E. faecalis 10C1 confirmed the presence of the active-site cysteine in all analyzed genes (Cys43/44) and showed similarity in the surrounding amino acid sequence. Thus, the NADH oxidase of all *Bifidobacterium* strains would be expected to have H_2_O-forming activity instead of H_2_O_2_-forming activity, as was previously suggested for B. longum subsp. *longum* NCC2705 (27). Overall, species that lacked a NOX homolog were highly diverse in terms of O_2_-sensitivity, including strains of the O_2_-hypersensitive species B. adolescentis and *B. angulatum* but also isolates from the digestive tract of bees. Therefore, the absence of a NOX homolog does not seem to be determinative of the O_2_ sensitivity of strains.

Several studies found a positive correlation between O_2_ tolerance of *Bifidobacterium* strains and the ability to detoxify H_2_O_2_ (20, 69, 70). Genomics and proteomics have revealed that bifidobacteria lack a gene for NADH peroxidase (35, 78) and that an alkyl hydroperoxide reductase subunit C (AhpC) and a thioredoxin reductase-like protein (TrxR), encoded next to AhpC, instead may be responsible for H_2_O_2_ decomposition (25, 27, 35, 78). Our results showed that in 78% of the analyzed species, all strains harbored both an AhpC and TrxR homolog (Fig. 1). No strain possessed only one of the genes, which reaffirms the assumption of their coactivity (Fig. S1). The presence of the AhpC-TrxR system in O_2_-sensitive strains, such as B. longum subsp. *infantis* ATCC 15697, suggests that in these strains the H_2_O_2_ detoxification activity provided by the system is insufficient to cope with H_2_O_2_ formation under aerobic conditions. Strains of the species *B. angulatum*, including *B. angulatum* DSM 20098, reported to be highly O_2_-sensitive (Table S3), *B. dentium*, *B. lemurum*, *B. subtile*, and some strains of B. adolescentis, including B. adolescentis ATCC 15703, which is also known to be highly O_2_ sensitive (Table S3), lacked both genes (Fig. 1 and Fig. S1). A second gene encoding a thioredoxin reductase (TrxR), which has been identified in B. longum subsp. *longum* NCC2705 and B. bifidum JCM1255, that lacks the N-terminal domain of the disulfide reductase AhpF and seems not to be induced by O_2_ stress (13, 25) was found to be highly conserved across genomes of species included in this study (Fig. 1).

Besides the AhpC-TrxR system, the bacterioferritin comigratory protein (BCP), first detected in B. longum subsp. *longum* NCC2705 (described as thioredoxin-dependent thiol peroxidase) (35) and induced in relatively H_2_O_2_-tolerant *B. animalis* subsp. *lactis* 01 upon oxidative stress (16), may contribute to thioredoxin-dependent H_2_O_2_-peroxidase activity in *Bifidobacterium* strains. In E. coli and Helicobacter pylori, BCP shows a substrate preference for linoleic acid hydroperoxide over H_2_O_2_ (79, 80), but the substrate specificity of BCP in bifidobacteria remains to be investigated. Homologs of BCP from B. longum subsp. *longum* NCC2705 were found in all strains of 55% of the studied species (Fig. 1). The analysis conducted in our study showed an overlap in the subset of strains that lacked the AhpC-TrxR system and strains that lacked a BCP, including all *B. angulatum* and *B. dentium* strains (Fig. 1) and some B. adolescentis strains, including B. adolescentis ATCC 15703 (Fig. S1). In addition, species of the *B. asteroides* group and *B. subtile* lacked a BCP homolog (Fig. 1).

**FIG 4:**
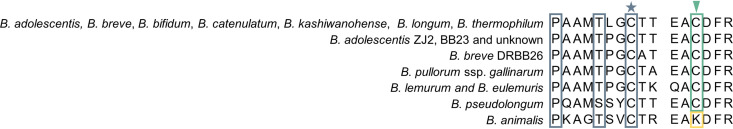
Active-site motif of BCP homologs in *Bifidobacterium* strains. ★, peroxidatic cysteine; ▾, resolving cysteine, which is absent from BCPs of *B. animalis*.

Since BCP from *B. animalis* and B. pseudolongum strains shows low sequence identity to the query gene (Fig. 1), the sequence motifs of the BCP gene products were further compared in a multiple-sequence alignment. In general, the active-site motif of peroxiredoxins (P-xxx-T/S-xx-C) includes a conserved cysteine (peroxidatic cysteine) that acts with the peroxide (R-O-O-R) to form a cysteine sulfenic acid (R-SOH) (75). Some peroxiredoxins contain a second cysteine (resolving) that can form a disulfide bond with R-SOH under the formation of H_2_O that is later reduced by a thiol-containing disulfide reductase system (75). In contrast, R-SOH in peroxiredoxins lacking a second cysteine form an intermolecular disulfide bond with a thiol of another protein or small molecule (75). The multiple-sequence alignment revealed that BCPs from *B. animalis* lacked a resolving cysteine in the active site, while the BCPs of all other species possess a second cysteine and share a motif (P-xxx-T/S-xx-C-xxxx-C) (Fig. 4). Besides the absence of the resolving cysteine in strains of three species, the motif around the catalytic cysteine in BCP showed additional variations across species (Fig. 4).

Additional ROS detoxification enzymes detected in bifidobacteria included the DNA-binding protein from starved cells (Dps) and the peptide-methionine sulfoxide reductase (MsrA/B). Dps protects cells from oxidative damage through binding genomic DNA and by sequestering free Fe^2+^ and H_2_O_2_ that could otherwise react in Fenton reactions, resulting in OH^·^ formation (81). MsrAB reduces methionine sulfoxide (resulting from the oxidation of methionine) back to methionine and thereby scavenges ROS (82). Strains of 27% of the studied species were found to lack a homolog of Dps (Fig. 1), including highly O_2_-sensitive strains of B. adolescentis and *B. angulatum* as well as relatively O_2_-tolerant strains of *B. thermophilum* (Table S3). In contrast, multiple B. longum and B. breve strains were found to possess a second copy of Dps.

All Dps-negative strains also lacked a gene encoding MsrAB (Fig. S1). MsrAB was further absent from nine additional strains of *B. dentium*, *B. choerinum*, and *B. pullorum* subsp. *gallinarum* and isolates from lemurs. Due to the various O_2_ sensitivities of strains lacking a Dps and MsrAB homolog, their sole absence appears not to be indicative of O_2_ sensitivity.

Overall, studying the prevalence of genes associated with the oxidative stress response in bifidobacterial genomes showed that the absence of single stress-associated gene products, such as Dps, AhpC, TrxR, and MsrAB, may not be decisive in the sensitivity of a strain to O_2_. Instead, the results indicated that the lack of multiple ROS-detoxifying genes, as observed for *B. angulatum* and some B. adolescentis strains, might cause exceptionally high sensitivity to O_2_. Strains of *B. dentium*, which have not been described as being particularly O_2_-sensitive, lacked the same ROS-detoxifying enzymes as *B. angulatum* and some B. adolescentis strains except for Dps (Fig. 1 and Fig. S1). Comparing the O_2_ tolerance of *B. dentium* with *B. angulatum* and B. adolescentis might provide some insights into the importance of Dps for survival under aerobic conditions. Moreover, comparing the O_2_ tolerance of B. adolescentis strains that were found to harbor AhpC, TrxR, and BCP with those that lacked these enzymes could validate the significance of these three ROS-detoxifying enzymes for O_2_ tolerance.

Surprisingly, O_2_-tolerant strains of the species *B. animalis* subsp. *lactis* harbored almost the same set of O_2_-scavenging and ROS-detoxifying enzymes as O_2_-sensitive strains of B. longum, B. breve, and B. bifidum (Fig. S1). Gene regulation, the ratio of different enzymes involved in the ROS-detoxification system (25), as well as the efficiency of NAD(P)H production driving the oxidative stress response may vary between these *Bifidobacterium* species. More knowledge on the functions of individual enzymes as well as on O_2_ tolerance of individual strains will need to be collected to allow further connections between genotypes and O_2_ sensitivity.

### Acid stress response.

Based on the upregulation and increased activity of the F_1_F_0_-ATP synthase upon acidic stress, the enzyme has been suggested to improve pH homeostasis by ATP-driven extrusion of protons (10, 11, 64, 83). As stated previously, the genes encoding F_1_F_0_-ATP synthase homologs were highly conserved across the analyzed species (Fig. 1), but its primary role might be different in putative respiring and nonrespiring strains.

The prevalence of nine additional genes associated with acid stress among bifidobacterial genomes was assessed in this study. Three of the genes, namely, polyphosphate kinase (PPK1), glutamate-cysteine ligase (GCL), and aminopeptidase PepP, were found to be highly conserved (Fig. 1). PPK1 is responsible for the formation of polyphosphate granules, which was found to contribute to acid stress resistance in *B. scardovii* JCM 12489 (14). Polyphosphate granules may promote stress resistance by serving as a reserve energy source and contributing to pH homeostasis (84). GCL and PepP were reported to be upregulated upon acid stress in B. longum strains (10, 11, 83). GCL catalyzes the formation of the glutathione precursor γ-glutamylcysteine. Since bifidobacteria lack a candidate gene encoding glutathione synthase (based on functional annotation information in NCBI GenBank), it has been suggested that γ-glutamylcysteine itself has a protective function under acid stress (11). The high degree of conservation of PepP across *Bifidobacterium* species, which may supply free amino acids for synthesis and repair of proteins under acidic conditions from peptides, agrees with the high degree of conservation observed for other members of the PQC system.

Oxalyl-coenzyme A (CoA) decarboxylase and formyl-CoA transferase are responsible for the detoxification of oxalate, a strong acid present in plant-based food. The expression of both enzymes was found to be induced upon acid stress in the acid-tolerant *B. dentium* Bd1 (12). Our genomic analysis showed that oxalyl-CoA decarboxylase and formyl-CoA transferase homologs from *B. dentium* Bd1 were only present in strains from the three species in the B. pseudolongum group, in B. pseudocatenulatum strains, and in other *B. dentium* strains (Fig. 1). Strains of these species have previously been shown to be acid tolerant (Table S3). The detoxification of oxalate, which is further linked to the scavenging of a proton (85), may contribute to coping with acidic conditions. However, the presence of oxalyl-CoA decarboxylase and formyl-CoA transferase homologs can only explain their acid tolerance in the presence of oxalate.

Enzymes involved in amino acid metabolism in *Bifidobacterium* strains were proposed to contribute to pH homeostasis upon acid stress due to proton scavenging, including the glutamate decarboxylase (GadB) pathway. GadB catalyzes the conversion of glutamate to gamma-aminobutyrate (GABA), consuming an intracellular proton, while a glutamate/gamma-aminobutyrate antiporter (GadC) transports GABA out of the cell and exchanges it with another glutamate (86). Genes encoding GadC and GadB were found to be upregulated in *B. dentium* Bd1 when subjected to acid stress (12). Homologs of the two gene products from *B. dentium* Bd1 were only found in B. adolescentis, *B. angulatum*, and other *B. dentium* strains (Fig. 1). The ability to produce GABA and the prevalence of *gadB* and *gadC* genes in their genome also was described for *B. moukalabense*, *B. stercoris*, *B. merycicum*, and *B. ruminantium*, which are not included in this study (87). A B. longum subsp. *infantis* strain was suggested to be a GABA producer (88), but none of the 11 B. longum subsp. *infantis* strains examined here were found to have GadC or GadB homologs (Fig. S1).

In the acid-resistant water kefir isolates *B. aquikefiri* CCUG 67145^T^ and *B. tibiigranuli* TMW 2.2057^T^ (89), a glutamine-ABC transporter, asparagine synthetase, asparaginase, and asparagine permease have been suggested to form a pathway that contributes to pH homeostasis under acid conditions by ammonia formation, which can scavenge protons in the cytoplasm (18). Like the genes encoding the glutamate decarboxylase pathway, genes encoding asparaginase and asparagine synthetase were only found in B. adolescentis, *B. angulatum*, and *B. dentium* (Fig. 1), whereas a few strains of other species possess one of the two genes (Fig. S1). In contrast to strains of *B. dentium*, strains of B. adolescentis and *B. angulatum* are not known to be particularly acid tolerant (Table S3) (34, 61). Thus, assessing the presence of the genes of the glutamate decarboxylase pathway and the described asparagine synthetase/asparaginase pathway appears to be insufficient to infer acid tolerance.

### Bile stress response.

Bile salt hydrolases (BSHs), which catalyze the deconjugation of primary and secondary bile salts, have been extensively studied in the context of bile salt resistance of bifidobacteria. However, it remains unclear if and how the activity of BSHs contributes to higher bile stress resistance (90, 91). Several BSHs have been characterized in bifidobacteria (92–95), where they exhibit low sequence similarity among different species (96). BSH homologs were found in 94% of the strains analyzed in this study and showed high sequence variation (Fig. 1). The lowest sequence identity was detected in strains of *B. angulatum* (47%). In agreement with their natural environment, no BSH homolog was detected in isolates from the digestive tract of bees or isolates from lemurs. Interestingly, lemurs lack the ability to produce glycine-conjugated bile salts (97), which are commonly the preferred substrate of bifidobacterial BSH (93, 94, 98) and are associated with higher toxicity than the taurine-conjugated bile salts (99, 100). This suggests that the absence of a BSH-encoding gene in the genomes of isolates from lemurs is a result of habitat adaptation or that these strains colonize lemurs, as they cannot survive in other animals that produce glycine-conjugated bile salts. Moreover, no BSH homologs were detected in *B. pullorum* subsp. *gallinarum* CACC 514 and *B. scardovi* JCM 12489 (only representative of *B. scardovi* species) (Fig. S1); however, the biological significance of this remains unknown.

### Organic solvent stress response.

Little is known about the stress response in bifidobacteria to organic solvents. However, an oleate hydratase in B. breve NCFB 2258 has previously been suggested to contribute to tolerance to butanol (2). Homologs of the oleate hydratase from B. breve NCFB 2258 were detected in strains of most species (Fig. 1). Strains lacking homologs were strains of the *B. asteroides* group, *B. subtile*, *B. eulemuris* DSM 100216 (only representative of *B. eulemuris* species), B. longum BORI and CCUG 30698, and *B. animalis* subsp. *lactis* ATCC 27673, which has already been described as having a genetic content distinct from that of other strains of the species (101).

### Putative transcriptional regulators of stress response.

In addition to the regulatory mechanism described for the PQC and DNA repair systems, the presence of homologs of additional stress-associated transcriptional regulators identified in *Bifidobacterium* strains was investigated (9, 13).

All strains analyzed in this study possessed the housekeeping sigma factor HrdB (homolog of RpoD) (Fig. 1). Moreover, all strains, except for *B. pullorum* subsp. *gallinarum* CACC 514, *B. coryneforme* LMG18911, and *B. indicum* LMG 11587, also possessed a homolog of an alternative sigma factor, extracytoplasmic function (ECF) RNA polymerase sigma factor RpoE, in their genomes (RpoE_1 in Fig. 1). In some strains included in the study, the RpoE was shorter (211 to 227 amino acids) than the query gene product from B. breve UCC2003 (253 amino acids), resulting in a query coverage between 71% and 79% (Data Set S1). Alternative sigma factors are known to control specialized regulons, e.g., regulons of stress response genes (102). However, very little is known about the function of RpoE in the stress response in bifidobacteria. The presence of RpoE has previously only been described in the genomes of B. breve UCC2003 and B. longum strains (13, 47, 50, 103), and which genes are controlled by RpoE still needs to be investigated, e.g., by applying DNase footprinting. Due to the absence of a consensus binding motif in the promoter region of the *hsp20* gene, it has been suggested that RpoE regulates the expression of Hsp20 (30). However, this hypothesis is contradicted by the observation that a negative effector of RpoE is upregulated in B. longum subsp. *longum* NCC2705 upon heat stress and the expression of *hsp20* is induced simultaneously (13). Moreover, whereas RpoE was found to be widely conserved in our analysis, Hsp20 homologs were only found in a subset of species, mainly representing isolates from humans (Fig. 1).

A second copy of RpoE was identified in 36 strains from B. breve and B. longum and in isolates from bees, including *B. asteroides*, *B. coryneforme*, and *B. indicum* (RpoE_2 in Fig. 1). Interestingly, all strains possessing a second copy of RpoE were also found to harbor a gene encoding a homolog of the putative SOD from B. longum subsp. *infantis* ATCC 15697. In B. longum subsp. *infantis*, the gene encoding RpoE (Blon_1402) is close to the gene encoding the putative SOD (Blon_1406), so it is conceivable that RpoE plays a role in transcriptional regulation of the putative SOD. In general, the presence of RpoE in all strains and a second copy in some strains indicates an important role of the sigma factor in transcriptional regulation.

In a previous study, two general types of WhiB-type regulators, WhiB2 and WblE, were detected in *Bifidobacterium* strains (9). WhiB-like proteins are thought to function as transcriptional regulators of major cellular processes, including stress responses, in *Actinobacteria* (104). While *wblE* orthologs have been suggested to be highly conserved, no *whiB2* orthologs have been identified in *B. animalis* subsp. *lactis* and *B. gallicum* strains (9). In addition, some strains have been found to have additional *whiB-like* genes with various lengths and low sequence similarity and were suggested to provide an additional benefit for sensing external signals (9). In B. longum B379M, the expression of *wblE* was induced upon exposure to various stressors, whereas no stress-induced transcription was observed for *whiB2* (9). In contrast, B. breve UCC2003 showed upregulation of the *whiB2* gene upon osmotic stress (see supplemental material in reference 24).

The analysis conducted in our study verified the presence of a WblE homolog in all strains of the analyzed species and revealed a high conservation in terms of its protein sequence (minimum of 88% identity). Moreover, the results confirmed that strains of *B. animalis* lack a WhiB2 homolog (Fig. 1). In addition, no WhiB2 homologs could be detected in genomes of other animal isolates (Fig. 1). Homologs of a second WhiB-like protein from B. longum subsp. *longum* NCC2705 (9, 35) were found in genomes of some strains of the B. longum group, B. catenulatum subsp. *kashiwanohense* and *B. kashinowehense* (Whib-like protein_1 in Fig. S1). Homologs with very low identity (43% to 50%) were detected in *B. subtile* and *B. pullorum* subsp. *gallinarum* (Fig. S1), suggesting that these WhiB-like proteins differ in function compared to the protein in B. longum subsp. *longum* NCC2705. Homologs of two additional WhiB-like proteins of the well-characterized bee isolate *B. asteroides* PRL2011 across strains of this study were assessed (Whib-like protein_2 and Whib-like protein_3). Whereas high-identity homologs were found for one of the genes in strains of various phylogenetic groups, the second appeared to be present only in *B. asteroides* and *B. thermophilum* (Fig. 1 and Fig. S1). Overall, the role of WhiB-like proteins in stress responses remains to be further investigated; however, the diverse prevalence of WhiB-like proteins in bifidobacteria suggests that their role in the transcriptional regulation of genes has variable impacts among strains and species.

### Conclusions.

Our analysis revealed multiple differences in the stress-associated genetic makeup of different *Bifidobacterium* species as well as some variations among strains of the same species, e.g., B. adolescentis. While genes encoding molecular players of the PQC and DNA repair systems were found to be highly conserved across species, the regulatory mechanisms of these systems might differ between phylogenetic groups. The presence/absence pattern of genes linked to oxidative stress response could not fully explain variations in the O_2_ tolerance across species, suggesting that their expression level upon oxidative stress influences O_2_ sensitivity. However, the absence of several ROS-detoxifying enzymes in strains of *B. angulatum* and B. adolescentis most likely contributes to their O_2_ sensitivity. Species-dependent genes associated with acid stress response might be a result of adaptation to natural habitats. Similarly, the presence of a gene encoding a bile salt hydrolase in human and mammalian isolates appeared highly linked to the presence of bile salts in their natural habitat. Moreover, the distribution of putative regulators of various stress responses varied across species, indicating that the involvement of different sets of regulators contributes to the highly diverse stress physiology of *Bifidobacterium* species.

In conclusion, mining bifidobacterial genomes for homologs of genes associated with various stress responses and their regulatory elements provided new insights into the molecular mechanisms underlying their diverse stress physiology. The obtained results lay out the foundation for hypothesis generation in future studies. However, systematic assessment of the effect of various stressors on growth and survival of sequenced *Bifidobacterium* strains as well as the identification of the biological function of stress-induced genes is required to obtain stronger correlations between the presence of stress-associated genes and stress tolerance of different strains.

## MATERIALS AND METHODS

### Identification of representative stress-associated genes.

Previous studies on stress response in bifidobacteria were consulted to identify representative genes that have been implicated in the stress response of bifidobacteria (see Table S1 in the supplemental material) (2–32, 35, 59, 64, 65). For the genomic analysis, the protein sequence of each representative gene was extracted from the genome sequence of a *Bifidobacterium* strain in which it was proposed to be involved in stress responses. Moreover, NCBI GenBank was searched for bifidobacterial genes annotated as superoxide dismutase and l-aspartate oxidase.

### Selection of strains.

All publicly available complete genome sequences of *Bifidobacterium* strains were retrieved from the NCBI RefSeq database, resulting in 171 genomes representing 22 out of 54 currently recognized *Bifidobacterium* species (Table 1) (61). All strains included in the study are listed in Table S2. Including only complete RefSeq genomes was intended to minimize the effect of fragmented genes in incomplete draft genomes, which might be missed with strict homology search parameters. The genome sequences were retrieved using the ncbi-genome-download package (K. Blin, https://github.com/kblin/ncbi-genome-download) with the arguments “–genera Bifidobacterium –section refseq –assembly-levels complete.” The RefSeq genomes include structural and functional annotations computed through NCBI’s PGAP.

### Homology search.

The proteomes of the 171 *Bifidobacterium* strains were searched for homologs of the representative stress-associated gene products using the DIAMOND BLASTp algorithm (v2.0.9.147) (105). For each genome, only the hit with the highest score for a given query was selected. However, information on the presence of additional hits was also collected. If a gene was the best hit for multiple query genes, only the best hit was retrieved. Default settings were used for most parameters, including E value (0.001), while applying a cutoff of 40% sequence identity across 70% of the protein sequence length. The maximum number of returned hits was set to 1,000 (default, 25). The results of the homology search were summarized by identifying the sequence identity of the highest hit for each query sequence in each bifidobacterial genome. Since there was only very limited variation in the presence of most stress-associated genes across strains of the same species (Fig. S1), the results were aggregated by species. This was done by calculating the median sequence identity of the best hit for a given query across all strains of each species. To visualize the presence of stress-associated genes in an evolutionary context, the phylogeny of the strains included in the study was constructed using the housekeeping gene *dnaA*. A maximum likelihood phylogeny of the *dnaA* sequences was constructed in CLC Genomics Workbench 20.0 with default settings, using the *dnaA* sequence from Gardnerella vaginalis (WP_004114028.1) as an outgroup. *Bifidobacterium* species were assigned to the previously suggested phylogenetic groups, including B. adolescentis, *B. asteroides*, *B. boum*, B. longum, B. pseudolongum, and *B. pullorum*, except for the strains of six species that have not yet been unambiguously assigned to any phylogenetic group (37, 40).

### Genome-wide *in silico* screening for known binding motifs.

The genomes of *B. animalis* subsp. *lactis* BB-12, B. adolescentis ATCC 15703, and B. longum subsp. *longum* NCC2705 were screened for the presence of previously proposed consensus sequences of the three transcriptional regulators HspR, HrcA, and ClgR (24). The online version of FIMO was used for the analysis (53). The threshold for the *P* value (probability of a random sequence of the same length as the motif matching the position of the sequence with as good or better score) was set to 0.0001, and the q-value (false discovery rate if the occurrence is accepted as significant) was set to 0.01 (53).

### Multiple-sequence alignments.

Multiple-sequence alignments were run in CLC Genomics Workbench 20.0 with default settings, using the progressive alignment mode “very accurate (slow).”

### Search for HemW-specific motifs in HemN homologs of *Bifidobacterium* strains.

The protein sequences of HemN in B. longum subsp. *longum* NCC2705, *B. animalis* subsp. *lactis* BB-12, and *B. asteroides* PRL2011 were compared to the protein sequence of HemW in Lactococcus lactis through multiple-sequence alignment and were subsequently screened for conserved amino acid residues specific for HemW-like proteins, including (i) His^134^, (ii) H^184^VxxYxLxLE, (iii) Y^234^ExS, and (iv) H^248^NxxYW (72).

### Data availability.

We declare that all the data supporting the work are available within the paper and its supplemental material.
