# Supplementary material for: Genome-Wide Assessment of Stress-Associated Genes in Bifidobacteria
Source: Appl Environ Microbiol. 2022 Mar 21;88(7):e02251-21. doi: 10.1128/aem.02251-21 (PMC9004370; doi:10.1128/aem.02251-21)
Supplement: Supplemental file 1 — Fig. S1 and Tables S1 to S3. Download aem.02251-21-s0001.pdf, PDF file, 0.4 MB [file aem.02251-21-s0001.pdf]

Supplementary material

Genome-wide assessment of stress-associated genes in bifidobacteria

Marie Schöpping[a,b], Tammi Vesth[a], Kristian Jensen[a], Carl Johan Franzén[b], Ahmad A. Zeidan[a,*]

Systems Biology, Discovery, R&D, Chr. Hansen A/S, 2970 Hørsholm, Denmark[a]; Division of Industrial Biotechnology, Department of Biology and Biological Engineering, Chalmers University of Technology, 41296 Gothenburg, Sweden[b]

[*] Corresponding author: DKAHZE@chr-hansen.com

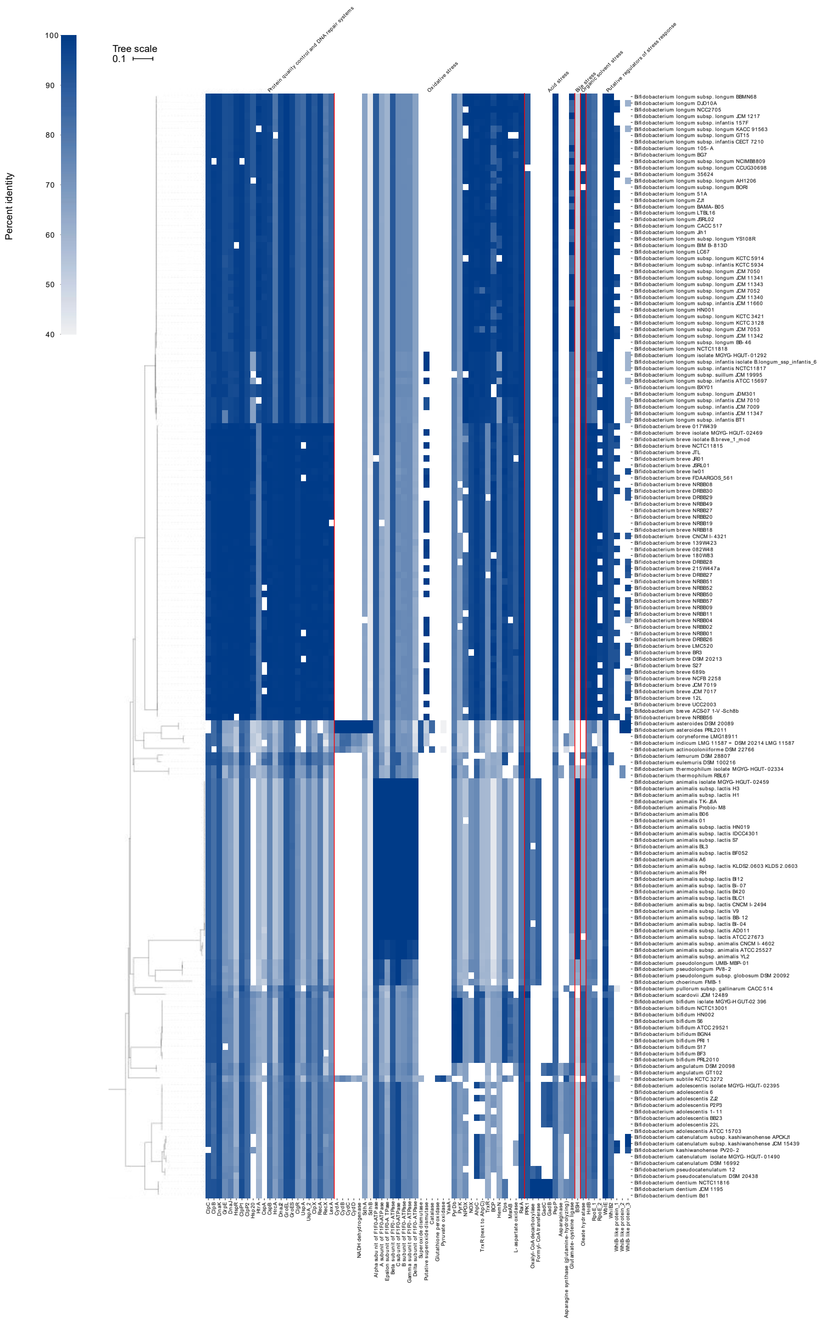

FIG S1: Heat map representing the presence and median sequence identity of the best hit of 76 stress-associated genes from 171 *Bifidobacterium* strains. The protein sequence homology was assessed using DIAMOND BLASTp. Default settings were used for most parameters including E-value (0.001), while applying a cutoff of 40% sequence identity across 70% of the protein sequence length. The maximum likelihood phylogeny tree was constructed using CLC Genomics Workbench. The tree scale gives the average number of substitutions per site. The sequence identity and coverage of each homolog can be found in Data S1.

Table S1: Stress-associated gene products included in the study. Genes which have been suggested to form an operon in the genome of *Bifidobacterium* strains are marked in the column 'Operon'.

Stressor	Protein name [a)]	PGAP annotation	Strain	RefSeq Identifier	Length (aa [b)])	Operon	Reference
**Protein quality control and DNA repair systems (Heat stress)**	ClpC	ATP-dependent Clp protease ATP-binding subunit	*B. breve* UCC2003	WP_003830303.1	869		(1)
	ClpB	ATP-dependent chaperone ClpB	*B. breve* UCC2003	WP_015439276.1	889		(2)
	DnaK	Molecular chaperone DnaK	*B. breve* UCC2003	WP_014483299.1	626	**Operon**	(3)
	GrpE	Nucleotide exchange factor GrpE	*B. breve* UCC2003	WP_003827988.1	227		(3)
	DnaJ	DnaJ domain containing protein	*B. breve* UCC2003	WP_003827989.1	337		(3)
	HspR, Heat shock protein repressor	helix-turn-helix transcriptional regulator	*B. breve* UCC2003	WP_003827990.1	195		(3, 4)
	ClpP1	ATP-dependent Clp protease proteolytic subunit	*B. breve* UCC2003	WP_003833421.1	207	**Operon**	(5)
	ClpP2	ATP-dependent Clp protease proteolytic subunit	*B. breve* UCC2003	WP_014483863.1	227		(5)
	Hsp20	Hsp20/ alpha crystallin family protein	*B. breve* UCC2003	WP_015438220.1	167		(6)
	HtrA	Trypsin-like peptidase domain-containing protein	*B. longum* ssp. *longum* NCC2705	WP_011068543.1	675		(7)
	CspA	Cold-shock protein	*B. breve* UCC2003	WP_003830292.1	79		(8)
	CspB	Cold shock domain-containing protein	*B. breve* UCC2003	WP_003830298.1	129		(1)
	HrcA	Heat-inducible transcriptional repressor HrcA	*B. breve* UCC2003	WP_015438802.1	365	**Operon**	(9)
	DnaJ2	Molecular chaperone DnaJ	*B. breve* UCC2003	WP_015438803.1	381		(9)
	GroEL	Chaperonin GroEL	*B. breve* UCC2003	WP_003830293.1	541		(8)
	GroES	Co-chaperone GroES	*B. breve* UCC2003	WP_003829844.1	97		(8)
	ClgR	Helix-turn-helix transcriptional regulator	*B. breve* UCC2003	WP_080562228.1	172		(1, 5)
	UspA, universal stress protein (next to *clpC* gene)	Universal stress protein	*B. breve* UCC2003	WP_014483540.1	321		(1)

	UspA_2, universal stress protein	Universal stress protein	*B. breve* UCC2003	WP_014484218.1	345		(10)
	ClpX	ATP-dependent Clp protease ATP-binding subunit ClpX	*B. breve* UCC2003	WP_003833417.1	453		(5)
	RecA, recombinase	Recombinase RecA	*B. breve* UCC2003	WP_015438912.1	392		(11)
	RecX, recombination regulator	RecX family transcriptional regulator	*B. breve* UCC2003	WP_015438911.1	199		(11)
	LexA, regulator	Transcriptional repressor LexA	*B. breve* UCC2003	WP_003830434.1	237		(11)
**Oxidative Stress**	CydA, cytochrome d oxidase subunit I	Cytochrome ubiquinol oxidase subunit I	*B. asteroides* PRL2011	WP_015021329.1	497	**Operon**	(12)
	CydB, cytochrome d oxidase subunit II	Cytochrome d ubiquinol oxidase subunit II	*B. asteroides* PRL2011	WP_015021326.1	350		
	CydC, cytochrome ABC-type transporter protein	Thiol reductant ABC exporter subunit CydC	*B. asteroides* PRL2011	WP_099327409.1	603		
	CydD, cytochrome ABC-type transporter protein	ABC transporter ATP-binding protein/permease	*B. asteroides* PRL2011	WP_099327362.1	596		
	NADH dehydrogenase	NAD(P)/FAD-dependent oxidoreductase	*B. asteroides* PRL2011	WP_015021324.1	391		(12)
	SdhA, succinate dehydrogenase complex A	FAD-dependent oxidoreductase	*B. asteroides* PRL2011	WP_033511903.1	633		(12)
	SdhB, succinate dehydrogenase complex B	4Fe-4S dicluster domain-containing protein	*B. asteroides* PRL2011	WP_033511913.1	381		(12)
	Alpha subunit of F_1F_0-ATPase, (*atpA*)	F0F1 ATP synthase subunit alpha	*B. animalis* ssp. *lactis* DSM 10140	WP_004219176.1	546	**Operon**	(13)
	A subunit of F_1F_0-ATPase, (*atpB*)	F0F1 ATP synthase subunit A	*B. animalis* ssp. *lactis* DSM 10140	WP_004219170.1	270		
	Epsilon subunit of F_1F_0-ATPase, (*atpC*)	F0F1 ATP synthase subunit epsilon	*B. animalis* ssp. *lactis* DSM 10140	WP_004219181.1	102		
	Beta subunit of F_1F_0-ATPase, (*atpD*)	F0F1 ATP synthase subunit beta	*B. animalis* ssp. *lactis* DSM 10140	WP_004219179.1	495		
	C subunit of F_1F_0-ATPase, (*atpE*)	F0F1 ATP synthase subunit C	*B. animalis* ssp. *lactis* DSM 10140	WP_004219171.1	76		
	B subunit of F_1F_0-ATPase, (*atpF*)	F0F1 ATP synthase subunit B	*B. animalis* ssp. *lactis* DSM 10140	WP_004219173.1	175		
	Gamma subunit of F_1F_0-ATPase, (*atpG*)	F0F1 ATP synthase subunit gamma	*B. animalis* ssp. *lactis* DSM 10140	WP_004219178.1	306		

	Delta subunit of F_1F_0-ATPase, (*atpH*)	F0F1 ATP synthase subunit delta	*B. lactis* DSM 10140	WP_004269232.1	277		
	Superoxide dismutase	Superoxide dismutase	*B. xylocopae* XV2	WP_113852633.1	206		(14)
		Putative superoxide dismutase	*B. longum* ATCC 15697	WP_003829426.1	132		
	Catalase	Catalase	*B. xylocopae* XV2	WP_113853686.1	480		(14)
	Glutathione peroxidase	Glutathione peroxidase	*B. tibiigranuli* TMW 2.2057	WP_152581073.1	188		(15)
	Pyruvate oxidase	Pyruvate oxidase	*B. tibiigranuli* TMW 2.2057	WP_152580610.1	615		(12, 15)
	YaaA, peroxide stress protein	Peroxide strass protein YaaA	*B. tibiigranuli* TMW 2.2057	WP_152581620.1	249		(15)
	PyrDb, dihydroorotate dehydrogenase	Dihydroorotate dehydrogenase	*B. bifidum* ATCC 29521	WP_021648026.1	320	**Operon**	(16)
	PyrK, dihydroorotate dehydrogenase electron transfer unit	Dihydroorotate dehydrogenase electron transfer unit	*B. bifidum* ATCC 29521	WP_003813104.1	276		
	NPOX, H_2O_2 forming NADPH oxidase	Nitroreductase family protein	*B. longum* ssp. *infantis* ATCC 15697	WP_012578666.1	254		(17)
	NOX, NADH oxidase	FAD-dependent oxidoreductase	*B. longum* NCC2705	WP_011068710.1	448		(18, 19)
	AhpC, alkyl hydroperoxide reductase subunit C	Peroxiredoxin	*B. longum* NCC2705	WP_007056578.1	187		(17, 19–25)
	TrxR (next to AhpC), thioredoxin reductase	FAD-dependent oxidoreductase	*B. longum* NCC2705	WP_007056574.1	638		(19–25)
	TrxR, thioredoxin reductase	Thioredoxin-disulfide reductase	*B. longum* NCC2705	WP_012472072.1	339		(22, 23)
	BCP, bacterioferritin comigratory protein,	Peroxiredoxin	*B. longum* NCC2705	WP_011068154.1	195		(20, 26)
	HemN, oxygen-independent coproporphyrinogen III oxidase	Radical SAM family heme chaperone HemW	*B. longum* NCC2705	WP_008783712.1	469		(18, 19)
	Dps, DNA-binding ferritin-like protein	DNA starvation/stationary phase protection protein	*B. longum* BBMN68	WP_007051742.1	159		(27)
	MsrAB, peptide-methionine sulfoxide reductase	Peptide-methionine (R)-S-oxide reductase MsrB	*B. longum* NCC2705	WP_162899813.1	324		(20, 26)
	L-aspartate oxidase	L-aspartate oxidase	*B. longum* NCC2705	WP_011068019.1	543		

	S30EA, also called ribosome-associated protein Y	Ribosome-associated translation inhibitor RaiA	*B. longum* BBMN68	WP_007054480.1	220		(27)
**Acid Stress**	PPK1, polyphosphate kinase	RNA degradosome polyphosphate kinase	*B. scardovii* JCM 12489	WP_033519427.1	745		(28)
	Oxalyl-CoA decarboxylase	Oxalyl-CoA decarboxylase	*B. dentium Bd1*	WP_003838690.1	589		(29)
	Formyl-CoA transferase	Formyl-CoA transferase	*B. dentium* Bd1	WP_012902505.1	436		(29)
	GadC, glutamate-γ-aminobutyrate antiporter	Amino acid permease	*B. dentium* Bd1	WP_003839068.1	470		(29)
	GadB, glutamate decarboxylase	Glutamate decarboxylase	*B. dentium* Bd1	WP_175279284.1	473		(29)
	PepP	Aminopeptidase P family protein	*B. longum* BBMN68	WP_007052570.1	531		(30)
	Asparaginase	Asparaginase	*B. tibiigranuli* TMW 2.2057	WP_152580025.1	335		(15)
	Asparagine synthase (glutamine-hydrolyzing)	Asparagine synthase (glutamine-hydrolyzing)	*B. tibiigranuli* TMW 2.2057	WP_152580942.1	627		(15)
	GCL, Glutamate-cysteine ligase	Gamma-glutamylcysteine synthetase	*B. longum BBMN68*	WP_013410698.1	425		(31)
**Organic solvent stress**	Oleate hydratase, also annotated as myosin-cross reactive antigen	Oleate hydratase	*B. breve* NCFB 2258	WP_015438992.1	625		(32, 33)
**Bile Stress**	BSH, bile salt hydrolase	Choloylglycine hydrolase	*B. animalis* ssp. *lactis* BB-12	WP_004217908.1	314		(34)
**Putative regulator of stress response**	HrdB, principal sigma factor (homolog of RpoD)	RNA polymerase sigma factor	*B. breve* UCC2003	WP_015438903.1	472		(11)
	RpoE_1	Sigma-70 family RNA polymerase sigma factor	*B. breve* UCC2003	WP_038426821.1	253		(6)
	RpoE_2	RNA polymerase sigma factor	*B. breve* UCC2003	WP_015438834.1	183		(6)
	WblE	WhiB family transcriptional regulator	*B. longum* NCC2705	WP_003835265.1	92		(35)
	WhiB2	WhiB family transcriptional regulator	*B. longum* NCC2705	WP_007051901.1	99		(35)

	WhiB-like protein_1	WhiB family transcriptional regulator	*B. longum* NCC2705	WP_011068092.1	158		(35)
	WhiB-like protein_2	WhiB family transcriptional regulator	*B. asteroides* PRL2011	WP_033511473.1	105		
	WhiB-like protein_3	WhiB family transcriptional regulator	*B. asteroides* PRL2011	WP_015021885.1	195		

[a] Protein names are given according to their denotation in the references. Generally accepted abbreviations for the proteins are given. If multiple proteins shared their name, they were numbered consecutively. [b] aa: amino acids.

References

1. Ventura M, Fitzgerald GF, van Sinderen D. 2005. Genetic and transcriptional organization of the clpC locus in *Bifidobacterium breve* UCC 2003. Appl Environ Microbiol 71:6282–91.

2. Ventura M, Kenny JG, Zhang Z, Fitzgerald GF, van Sinderen D. 2005. The *clpB* gene of *Bifidobacterium breve* UCC 2003: Transcriptional analysis and first insights into stress induction. Microbiology 151:2861–2872.

3. Ventura M, Zink R, Fitzgerald GF, Sinderen D Van. 2005. Gene structure and transcriptional organization of the *dnaK* operon of *Bifidobacterium breve* UCC 2003 and application of the operon in bifidobacterial tracing. Appl Environ Microbiol 71:487–500.

4. Berger B, Moine D, Mansourian R, Arigoni F. 2010. HspR mutations are naturally selected in *Bifidobacterium longum* when successive heat shock treatments are applied. J Bacteriol 192:256–263.

5. Ventura M, Zhang Z, Cronin M, Canchaya C, Kenny JG, Fitzgerald GF, Sinderen D Van. 2005. The ClgR protein regulates transcription of the *clpP* operon in *Bifidobacterium breve* UCC 2003. J Bacteriol 187:8411–8426.

6. Ventura M, Canchaya C, Zhang Z, Fitzgerald GF, Van Sinderen D. 2007. Molecular characterization of *hsp20*, encoding a small heat shock protein of *Bifidobacterium breve* UCC2003. Appl Environ Microbiol 73:4695–4703.

7. Savijoki K, Suokko A, Palva A, Valmu L, Kalkkinen N, Varmanen P. 2005. Effect of heat-shock and bile salts on protein synthesis of *Bifidobacterium longum* revealed by [^{35}S]methionine labelling and two-dimensional gel electrophoresis. FEMS Microbiol Lett 248:207–215.

8. Ventura M, Canchaya C, Zink R, Fitzgerald GF, Sinderen D Van. 2004. Characterization of the *groEL* and *groES* loci in *Bifidobacterium breve* UCC 2003: Genetic, transcriptional, and phylogenetic analyses. Appl Environ Microbiol 70:6197–6209.

9. Ventura M, Canchaya C, Bernini V, Del Casale A, Dellaglio F, Neviani E, Fitzgerald GF, Van Sinderen D. 2005. Genetic characterization of the *Bifidobacterium breve* UCC 2003 *hrcA* locus. Appl Environ Microbiol 71:8998–9007.

10. Ruiz L, Zomer A, O'Connell-Motherway M, van Sinderen D, Margolles A. 2012. Discovering novel bile protection systems in *Bifidobacterium breve* UCC2003 through functional genomics. Appl Environ Microbiol 78:1123–1131.

11. Zomer A, Fernandez M, Kearney B, Fitzgerald GF, Ventura M, Van Sinderen D. 2009. An interactive regulatory network controls stress response in *Bifidobacterium breve* UCC2003. J Bacteriol 191:7039–7049.

12. Foroni E, Duranti S, Serafini F, Viappiani A, Strati F, Ferrarini A, Delledonne M, Henrissat B, Coutinho P, Fitzgerald GF, Margolles A, Sinderen D Van, Ventura M. 2012. *Bifidobacterium asteroides* PRL2011 genome analysis reveals clues for colonization of the insect gut. PLoS One 7:1–14.

13. Ventura M, Canchaya C, van Sinderen D, Fitzgerald GF, Zink R. 2004. *Bifidobacterium lactis* DSM 10140: identification of the *atp* (*atpBEFHAGDC*) operon and analysis of its genetic structure, characteristics, and phylogeny. Appl Environ Microbiol 70:3110–3121.

14. Alberoni D, Gaggìa F, Baffoni L, Modesto MM, Biavati B, Di Gioia D. 2019. *Bifidobacterium xylocopae* sp. nov. and *Bifidobacterium aemilianum* sp. nov., from the carpenter bee (*Xylocopa violacea*) digestive tract. Syst Appl Microbiol 42:205–216.

15. Eckel VPL, Vogel RF. 2020. Genomic and physiological insights into the lifestyle of *Bifidobacterium* species from water kefir. Arch Microbiol https://doi.org/10.1007/s00203-020-01870-7.

16. Kawasaki S, Satoh T, Todoroki M, Niimura Y. 2009. *b*-type dihydroorotate dehydrogenase is purified as a H_2O_2-forming NADH oxidase from *Bifidobacterium bifidum*. Appl Environ Microbiol 75:629–636.

17. Tanaka K, Satoh T, Kitahara J, Uno S, Nomura I, Kano Y, Suzuki T, Niimura Y, Kawasaki S. 2018. O_2-inducible H_2O_2-forming NADPH oxidase is responsible for the hyper O_2 sensitivity of *Bifidobacterium longum* subsp. *infantis*. Sci Rep 8:1–10.

18. Zuo FL, Yu R, Khaskheli GB, Ma HQ, Chen LL, Zeng Z, Mao AJ, Chen SW. 2014. Homologous overexpression of alkyl hydroperoxide reductase subunit C (*ahpC*) protects *Bifidobacterium longum* strain NCC2705 from oxidative stress. Res Microbiol 165:581–589.

19. Ruiz L, Gueimonde M, Patricia RM, Ribbera A, de los Reyes-Gavilán CG, Ventura M, Margolles A, Sánchez B. 2012. Molecular clues to understand the aerotolerance phenotype of *Bifidobacterium animalis* subsp. *lactis*. Appl Environ Microbiol 78:644–650.

20. Zhang J, Wang S, Zeng Z, Qin Y, Li P. 2019. The complete genome sequence of *Bifidobacterium animalis* subsp. *lactis* 01 and its integral components of antioxidant defense system. 3 Biotech 9:352.

21. Zuo F, Yu R, Xiao M, Khaskheli GB, Sun X, Ma H, Ren F, Zhang B, Chen S. 2018. Transcriptomic

analysis of *Bifidobacterium longum* subsp. *longum* BBMN68 in response to oxidative shock. Sci Rep 8:17085.

22. Oberg TS, Ward RE, Steele JL, Broadbent R. 2013. Genetic and physiological responses of *Bifidobacterium animalis* subsp. *lactis* to hydrogen peroxide stress. J Bacteriol 195:3743–3751.

23. Klijn A, Mercenier A, Arigoni F. 2005. Lessons from the genomes of bifidobacteria. FEMS Microbiol Rev 29:491–509.

24. Oberg TS, Ward RE, Steele JL, Broadbent JR. 2015. Transcriptome analysis of *Bifidobacterium longum* strains that show a differential response to hydrogen peroxide stress. J Biotechnol 212:58–64.

25. Satoh T, Todoroki M, Kobayashi K, Niimura Y, Kawasaki S. 2019. Purified thioredoxin reductase from O_2-sensitive *Bifidobacterium bifidum* degrades H_2O_2 by interacting with alkyl hydroperoxide reductase. Anaerobe 57:45–54.

26. Schell MA, Karmirantzou M, Snel B, Vilanova D, Berger B, Pessi G, Zwahlen M-C, Desiere F, Bork P, Delley M, Pridmore RD, Arigoni F. 2002. The genome sequence of *Bifidobacterium longum* reflects its adaptation to the human gastrointestinal tract. Proc Natl Acad Sci 99:14422–14427.

27. Xiao M, Xu P, Zhao J, Wang Z, Zuo F, Zhang J, Ren F, Li P, Chen S, Ma H. 2011. Oxidative stress-related responses of *Bifidobacterium longum* subsp. *longum* BBMN68 at the proteomic level after exposure to oxygen. Microbiology 157:1573–1588.

28. Qian Y, Borowski WJ, Calhoon WD. 2011. Intracellular granule formation in response to oxidative stress in *Bifidobacterium*. Int J Food Microbiol 145:320–325.

29. Ventura M, Turroni F, Zomer A, Foroni E, Giubellini V, Bottacini F, Canchaya C, Claesson MJ, He F, Mantzourani M, Mulas L, Ferrarini A, Gao B, Delledonne M, Henrissat B, Coutinho P, Oggioni M, Gupta RS, Zhang Z, Beighton D, Fitzgerald GF, O'Toole PW, Van Sinderen D. 2009. The *Bifidobacterium dentium* Bd1 genome sequence reflects its genetic adaptation to the hum an oral cavity. PLoS Genet 5.

30. Jin J, Qin Q, Guo H, Liu S, Ge S, Zhang H, Cui J, Ren F. 2015. Effect of pre-stressing on the acid-stress response in *Bifidobacterium* revealed using proteomic and physiological approaches. PLoS One 10:1–14.

31. Jin J, Zhang B, Guo H, Cui J, Jiang L, Song S, Sun M, Ren F. 2012. Mechanism analysis of acid tolerance response of *Bifidobacterium longum* subsp. *longum* BBMN 68 by gene expression profile using

RNA-sequencing. PLoS One 7:e50777.

32. Rosberg-Cody E, Liavonchanka A, Göbel C, Ross RP, O'Sullivan O, Fitzgerald GF, Feussner I, Stanton C. 2011. Myosin-cross-reactive antigen (MCRA) protein from *Bifidobacterium breve* is a FAD-dependent fatty acid hydratase which has a function in stress protection. BMC Biochem 12.

33. O'Connell KJ, Motherway MOC, Hennessey AA, Brodhun F, Ross RP, Feussner I, Stanton C, Fitzgerald GF, Van Sinderen D. 2013. Identification and characterization of an oleate hydratase-encoding gene from *Bifidobacterium breve*. Bioengineered 4:37–41.

34. Garrigues C, Stuer-Lauridsen B, Johansen E. 2005. Characterisation of *Bifidobacterium animalis* subsp. *lactis* BB-12 and other probiotic bacteria using genomics, transcriptomics and proteomics. Aust J Dairy Technol 60:84–92.

35. Averina O V., Zakharevich N V., Danilenko VN. 2012. Identification and characterization of WhiB-like family proteins of the *Bifidobacterium* genus. Anaerobe 18:421–429.

Table S2: List of *Bifidobacterium* strains included in the study.

Strain name	NCBI assembly accession number
Bifidobacterium actinocoloniiforme, DSM 22766	GCF_001263395.1
Bifidobacterium adolescentis, 1-11	GCF_003030905.1
Bifidobacterium adolescentis, 22L	GCF_000737885.1
Bifidobacterium adolescentis, 6	GCF_003429385.1
Bifidobacterium adolescentis, ATCC 15703	GCF_000010425.1
Bifidobacterium adolescentis, BB23	GCF_000817995.1
Bifidobacterium adolescentis, P2P3	GCF_003856735.1
Bifidobacterium adolescentis, UHGG_MGYG-HGUT-02395	GCF_902386735.1
Bifidobacterium adolescentis, ZJ2	GCF_009832825.1
Bifidobacterium angulatum DSM 20098 = JCM 7096	GCF_001025155.1
Bifidobacterium angulatum, GT102	GCF_000966445.2
Bifidobacterium animalis subsp. *animalis* ATCC 25527	GCF_000260715.1
Bifidobacterium animalis subsp. *animalis*, CNCM I-4602	GCF_003671995.1
Bifidobacterium animalis subsp. *animalis*, YL2	GCF_001688645.2
Bifidobacterium animalis subsp. *lactis* AD011, AD011	GCF_000021425.1
Bifidobacterium animalis subsp. *lactis* ATCC 27673	GCF_000471945.1
Bifidobacterium animalis subsp. *lactis* B420	GCF_000277325.1
Bifidobacterium animalis subsp. *lactis* BB-12	GCF_000025245.2
Bifidobacterium animalis subsp. *lactis* Bi-07	GCF_000277345.1
Bifidobacterium animalis subsp. *lactis* Bl-04, ATCC SD5219	GCF_000022705.1
Bifidobacterium animalis subsp. *lactis* Bl12, Bl12	GCF_000414215.1
Bifidobacterium animalis subsp. *lactis* BLC1, BLC1	GCF_000224965.2
Bifidobacterium animalis subsp. *lactis* CNCM I-2494	GCF_000220885.1
Bifidobacterium animalis subsp. *lactis* KLDS2.0603	GCF_000816205.1
Bifidobacterium animalis subsp. *lactis* V9, V9	GCF_000092765.1
Bifidobacterium animalis subsp. *lactis*, 01	GCF_004135895.1
Bifidobacterium animalis subsp. *lactis*, A6	GCF_000817045.1
Bifidobacterium animalis subsp. *lactis*, BF052	GCF_000818055.1
Bifidobacterium animalis subsp. *lactis*, H1	GCF_016835115.1
Bifidobacterium animalis subsp. *lactis*, H3	GCF_016835135.1
Bifidobacterium animalis subsp. *lactis*, HN019	GCF_003606305.1
Bifidobacterium animalis subsp. *lactis*, IDCC4301	GCF_003428375.1
Bifidobacterium animalis subsp. *lactis*, Probio-M8	GCF_011770105.1
Bifidobacterium animalis subsp. *lactis*, S7	GCF_003390755.1
Bifidobacterium animalis subsp. *lactis*, TK-J6A	GCF_015377505.1
Bifidobacterium animalis, B06	GCF_008041995.1
Bifidobacterium animalis, BL3	GCF_002220485.1
Bifidobacterium animalis, RH	GCF_000695895.1

Bifidobacterium animalis, UHGG_MGYG-HGUT-02459	GCF_902387355.1
Bifidobacterium asteroides DSM 20089	GCF_002715865.1
Bifidobacterium asteroides PRL2011, PRL2011	GCF_000304215.1
Bifidobacterium bifidum ATCC 29521 = JCM 1255 = DSM 20456	GCF_001025135.1
Bifidobacterium bifidum BGN4, BGN4	GCF_000265095.1
Bifidobacterium bifidum PRL2010	GCF_000165905.1
Bifidobacterium bifidum S17	GCF_000164965.1
Bifidobacterium bifidum, BF3	GCF_001281345.1
Bifidobacterium bifidum, HN002	GCF_016838705.1
Bifidobacterium bifidum, NCTC13001	GCF_900637095.1
Bifidobacterium bifidum, PRI 1	GCF_002845845.1
Bifidobacterium bifidum, S6	GCF_003390735.1
Bifidobacterium bifidum, UHGG_MGYG-HGUT-02396	GCF_902386775.1
Bifidobacterium breve 1_mod	GCF_902167875.1
Bifidobacterium breve 12L, 12L	GCF_000568955.1
Bifidobacterium breve 689b, 689b	GCF_000569055.1
Bifidobacterium breve ACS-071-V-Sch8b	GCF_000213865.1
Bifidobacterium breve DSM 20213 = JCM 1192	GCF_001025175.1
Bifidobacterium breve JCM 7017	GCF_000568975.1
Bifidobacterium breve JCM 7019	GCF_000569015.1
Bifidobacterium breve NCFB 2258	GCF_000569035.1
Bifidobacterium breve S27	GCF_000569075.1
Bifidobacterium breve UCC2003	GCF_000220135.1
Bifidobacterium breve, 017W439	GCF_002838465.1
Bifidobacterium breve, 082W48	GCF_002838545.1
Bifidobacterium breve, 139W423	GCF_002838565.1
Bifidobacterium breve, 180W83	GCF_002838525.1
Bifidobacterium breve, 215W447a	GCF_002838485.1
Bifidobacterium breve, BR3	GCF_001281425.1
Bifidobacterium breve, CNCM I-4321	GCF_002838585.1
Bifidobacterium breve, DRBB26	GCF_002838225.1
Bifidobacterium breve, DRBB27	GCF_002838445.1
Bifidobacterium breve, DRBB28	GCF_002838505.1
Bifidobacterium breve, DRBB29	GCF_002838705.1
Bifidobacterium breve, DRBB30	GCF_002838725.1
Bifidobacterium breve, FDAARGOS_561	GCF_003813065.1
Bifidobacterium breve, JR01	GCF_009931415.1
Bifidobacterium breve, JSRL01	GCF_009498435.1
Bifidobacterium breve, JTL	GCF_013267755.1
Bifidobacterium breve, LMC520	GCF_001990225.1
Bifidobacterium breve, lw01	GCF_003860285.1

Bifidobacterium breve, NCTC11815	GCF_900637145.1
Bifidobacterium breve, NRBB01	GCF_002838245.1
Bifidobacterium breve, NRBB02	GCF_002838265.1
Bifidobacterium breve, NRBB04	GCF_002838285.1
Bifidobacterium breve, NRBB08	GCF_002838745.1
Bifidobacterium breve, NRBB09	GCF_002838325.1
Bifidobacterium breve, NRBB11	GCF_002838305.1
Bifidobacterium breve, NRBB18	GCF_002838605.1
Bifidobacterium breve, NRBB19	GCF_002838625.1
Bifidobacterium breve, NRBB20	GCF_002838645.1
Bifidobacterium breve, NRBB27	GCF_002838665.1
Bifidobacterium breve, NRBB49	GCF_002838685.1
Bifidobacterium breve, NRBB50	GCF_002838365.1
Bifidobacterium breve, NRBB51	GCF_002838405.1
Bifidobacterium breve, NRBB52	GCF_002838385.1
Bifidobacterium breve, NRBB56	GCF_002838425.1
Bifidobacterium breve, NRBB57	GCF_002838345.1
Bifidobacterium breve, UHGG_MGYG-HGUT-02469	GCF_902387425.1
Bifidobacterium catenulatum DSM 16992 = JCM 1194 = LMG 11043	GCF_001025195.1
Bifidobacterium catenulatum subsp. *kashiwanohense* JCM 15439 = DSM 21854	GCF_001042615.1
Bifidobacterium catenulatum subsp. *kashiwanohense*, APCKJ1	GCF_009684555.1
Bifidobacterium catenulatum,	GCF_902381755.1
Bifidobacterium choerinum, FMB-1	GCF_002761235.1
Bifidobacterium coryneforme, LMG18911	GCF_000737865.1
Bifidobacterium dentium Bd1, Bd1	GCF_000024445.1
Bifidobacterium dentium, JCM 1195 = DSM 20436	GCF_001042595.1
Bifidobacterium dentium, NCTC11816	GCF_900637175.1
Bifidobacterium eulemuris, DSM 100216	GCF_014898155.1
Bifidobacterium indicum, LMG 11587 = DSM 20214	GCF_000706765.1
Bifidobacterium kashiwanohense, PV20-2	GCF_000800455.1
Bifidobacterium lemurum, DSM 28807	GCF_014898175.1
Bifidobacterium longum subsp. *infantis* 157F	GCF_000196575.1
Bifidobacterium longum subsp. *infantis* ATCC 15697 = JCM 1222 = DSM 20088	GCF_000269965.1
Bifidobacterium longum subsp. *infantis*,	GCF_902167885.1
Bifidobacterium longum subsp. *infantis*, BT1	GCF_001281305.1
Bifidobacterium longum subsp. *infantis*, CECT 7210	GCF_001051015.2
Bifidobacterium longum subsp. *infantis*, JCM 11347	GCF_015102215.1
Bifidobacterium longum subsp. *infantis*, JCM 11660	GCF_015102035.1
Bifidobacterium longum subsp. *infantis*, JCM 7009	GCF_017299595.1
Bifidobacterium longum subsp. *infantis*, JCM 7010	GCF_017378625.1
Bifidobacterium longum subsp. *infantis*, KCTC 5934	GCF_014898135.1

Bifidobacterium longum subsp. *infantis*, NCTC11817	GCF_900637215.1
Bifidobacterium longum subsp. *longum* BB-46	GCF_021184065.1
Bifidobacterium longum subsp. *longum* BBMN68	GCF_000166315.1
Bifidobacterium longum subsp. *longum* DJO10A	GCF_000008945.1
Bifidobacterium longum subsp. *longum* GT15	GCF_000772485.1
Bifidobacterium longum subsp. *longum* JCM 1217	GCF_000196555.1
Bifidobacterium longum subsp. *longum* JDM301	GCF_000092325.1
Bifidobacterium longum subsp. *longum* KACC 91563	GCF_000219455.1
Bifidobacterium longum subsp. *longum* NCC2705	GCF_000007525.1
Bifidobacterium longum subsp. *longum*, 105-A	GCF_000829295.1
Bifidobacterium longum subsp. *longum*, 35624	GCF_001719085.1
Bifidobacterium longum subsp. *longum*, AH1206	GCF_001725985.1
Bifidobacterium longum subsp. *longum*, BORI	GCF_003342655.1
Bifidobacterium longum subsp. *longum*, BXY01	GCF_000730205.1
Bifidobacterium longum subsp. *longum*, CCUG30698	GCF_001446275.1
Bifidobacterium longum subsp. *longum*, JCM 11340	GCF_015101725.1
Bifidobacterium longum subsp. *longum*, JCM 11341	GCF_014898235.1
Bifidobacterium longum subsp. *longum*, JCM 11342	GCF_017357325.1
Bifidobacterium longum subsp. *longum*, JCM 11343	GCF_014900535.1
Bifidobacterium longum subsp. *longum*, JCM 7050	GCF_014898215.1
Bifidobacterium longum subsp. *longum*, JCM 7052	GCF_015100215.1
Bifidobacterium longum subsp. *longum*, JCM 7053	GCF_017357345.1
Bifidobacterium longum subsp. *longum*, KCTC 3128	GCF_017132775.1
Bifidobacterium longum subsp. *longum*, KCTC 3421	GCF_017357065.1
Bifidobacterium longum subsp. *longum*, KCTC 5914	GCF_014898115.1
Bifidobacterium longum subsp. *longum*, NCIMB8809	GCF_001446255.1
Bifidobacterium longum subsp. *longum*, NCTC11818	GCF_900637335.1
Bifidobacterium longum subsp. *longum*, YS108R	GCF_013393765.1
Bifidobacterium longum subsp. *suillum*, JCM 19995	GCF_017132755.1
Bifidobacterium longum, 51A	GCF_004936435.1
Bifidobacterium longum, BAMA-B05	GCF_008086305.1
Bifidobacterium longum, BG7	GCF_001293145.1
Bifidobacterium longum, BIM B-813D	GCF_014334075.1
Bifidobacterium longum, CACC 517	GCF_009931635.1
Bifidobacterium longum, HN001	GCF_016838685.1
Bifidobacterium longum, Jih1	GCF_011764605.1
Bifidobacterium longum, JSRL02	GCF_009738515.1
Bifidobacterium longum, LC67	GCF_014334375.1
Bifidobacterium longum, LTBL16	GCF_009728915.1
Bifidobacterium longum, UHGG_MGYG-HGUT-01292	GCF_902381625.1
Bifidobacterium longum, ZJ1	GCF_005406285.1

Bifidobacterium *pseudocatenulatum*, 12	GCF_003952825.1
Bifidobacterium *pseudocatenulatum*, DSM 20438 = JCM 1200 = LMG 10505	GCF_001025215.1
Bifidobacterium pseudo*longum* PV8-2	GCF_000800475.2
Bifidobacterium pseudolongum subsp. *globosum* DSM 20092	GCF_002706665.1
Bifidobacterium pseudolongum, UMB-MBP-1	GCF_002282915.1
Bifidobacterium pullorum subsp. *gallinarum*, CACC 514	GCF_004135085.1
Bifidobacterium scardovii, JCM 12489 = DSM 13734	GCF_001042635.1
Bifidobacterium subtile, KCTC 3272	GCF_014898195.1
Bifidobacterium thermophilum	GCF_902385935.1
Bifidobacterium thermophilum RBL67	GCF_000347695.1

Table S3: Data on the tolerance of *Bifidobacterium* strains to oxidative, heat, acid, and bile stress collected from previous studies. The strains marked in blue are included in the study.

Strain name	Oxidative stress	Heat stress	Acid stress	Bile stress
B. actinocoloniiforme DSM 22766	Growth under microaerophilic conditions (1)			
B. adolescentis ATCC 15703 = DSM 20083	O_2-hypersensitive (2) Loss of viability when exposed to aerobic conditions or H_2O_2 (2, 3)		Low tolerance (4)	
B. angulatum ATCC 27535 = DSM 20098	O_2-hypersensitive (more sensitive to O_2 than other *Bifidobacterium* strains) (5)			Bile salt hydrolase activity (6)
B. animalis ssp. *animalis* ATCC 25527 = DSM 20104 = JCM 1190	No loss of viability when exposed to aerobic conditions (20 h) (3). Less O_2-tolerant than *B. lactis* UR1 (7)		High tolerance (8)	Shows bile salt hydrolase activity and is less sensitive to deconjugated bile salts than other *Bifidobacterium* strains (6)
B. animalis ssp. *lactis* 01	High H_2O_2-tolerance (9). High antioxidant activity (10)			
B. animalis ssp. *lactis* BB-12	O_2-tolerant (11)	High tolerance (11)	High tolerance (4)	
B. animalis ssp. *lactis* BL-04	Intermediate intrinsic H_2O_2 tolerance (12)			
B. animalis ssp. *lactis* IPLA4549	O_2-tolerant (13)			
B. animalis ssp. *lactis* RH-1	Comparatively low intrinsic H_2O_2 tolerance (12)			
B. animalis ssp. *lactis* UR-1 = DSM 10140 = JCM 10602	Moderately O_2-tolerant (14)		High tolerance (8)	
B. aquikefiri CCUG 67145 = LMG 28769	O_2-tolerant (15)		High tolerance (16)	
B. asteroides JCM 8230 DSM 20089 = ATCC 25910	O_2-tolerant (17), growth under microaerophilic conditions (1)			No bile salt hydrolase activity (6)
B. asteroides PRL2011	O_2-tolerant; might respire under aerobic conditions (18)			
B. bifidum JCM 1255 = DSM 20456 = ATCC 29521	O_2-sensitive (19), however, no loss of viability when exposed to aerobic conditions (20 h) (3)		Low tolerance (8)	
B. bombi BluCI/TP		Growth at low temperatures (20)		
B. boum JCM 1211 = ATCC 279197	O_2-hypertolerant (19)			No bile salt hydrolase activity (6)
B. breve JCM 1192	O_2-sensitive (19)			

B. choerinum ATCC 27686	Slightly anaerobic (21), Moderately O_2-tolerant (11)	High tolerance (11)		
B. coryneforme LMG 18911 = DSM 20214 = ATCC 25911	Growth under microaerophilic conditions (1)			No bile salt hydrolase activity (6)
B. dentium Bd1			High tolerance, growth at pH 4 (22)	
B. dentium JCM 1195 = DSM 20436 = ATCC 27534				Bile salt hydrolase activity (6)
B. indicum LMG 11587 = DSM 20214 = ATCC 25912	Growth under microaerophilic conditions (1)			No bile salt hydrolase activity (6)
B. lemurum JCM 30168 = DSM 28807	Growth under microaerophilic conditions (23)			
B. longum BB-46	O_2-sensitive (24)		Low tolerance (4)	
B. longum BBMN68	O_2-hypersensitive, 3% O_2 reduces growth rate but not lethal (25)			
B. longum NCC2705	Intermediate intrinsic H_2O_2 tolerance (12)			
B. longum ssp. infantis ATCC 15697 = JCM 1222 = DSM 20088	O_2-hypersensitive (26), however, only minor loss of viability when exposed to aerobic conditions (20 h) (3)		Low tolerance (4, 8)	Bile salt hydrolase activity (6)
B. longum ssp. *longum* JCM 7052			Very low tolerance (8)	
B. longum ssp. longum JCM 1217	O_2-sensitive, 5% O_2 seems to have a growth-promoting effect on the strain (19)		Very low tolerance (8)	
B. minimum DSM 20102	O_2-tolerant (26)			
B. pseudolongum ssp. *globosum* DSM 20092 = ATCC 25865 = JCM 5820	Moderately O_2-tolerant (11)	High tolerance (11)		Bile salt hydrolase activity (6)
B. pseudolongum ssp. *longum* ATCC 25525				Bile salt hydrolase activity (6)
B. subtile KCTC 3272 = DSM 20096	No growth under microaerophilic conditions (1)			
B. thermophilum JCM 1207 = ATCC 25525	O_2-hypertolerant, growth of the strain was sometimes arrested in the presence of 20% O_2 (19)			Bile salt hydrolase activity (6)
B. thermophilum RBL67	O_2-tolerant (7)	High tolerance (7)	High tolerance (7)	
B. tibiigranuli TMW 2.2057	O_2-tolerant (16)		High tolerance (16)	

References

1. Alberoni D, Gaggìa F, Baffoni L, Modesto MM, Biavati B, Di Gioia D. 2019. *Bifidobacterium xylocopae* sp. nov. and *Bifidobacterium aemilianum* sp. nov., from the carpenter bee (*Xylocopa violacea*) digestive tract. Syst Appl Microbiol 42:205–216.

2. Shimamura S, Abe F, Ishibashi N, Miyakawa H, Yaeshima T, Araya T, Tomita M. 1992. Relationship between oxygen sensitivity and oxygen metabolism of *Bifidobacterium* species. J Dairy Sci 75:3296–3306.

3. Shin SY, Park JH. 1997. Activities of oxidative enzymes related with oxygen tolerance in *Bifidobacterium* sp. J Microbiol Biotechnol 7:356–359.

4. Vernazza CL, Gibson GR, Rastall RA. 2006. Carbohydrate preference, acid tolerance and bile tolerance in five strains of *Bifidobacterium*. J Appl Microbiol 100:846–853.

5. Scardovi V, Crociani F. 1974. *Bifidobacterium catenulatum*, *Bifidobacterium dentium*, and *Bifidobacterium angulatum*: three new species and their deoxyribonucleic acid homology relationships. Int J Syst Bacteriol 24:6–20.

6. Grill JP, Perrin S, Schneider F. 2000. Bile salt toxicity to some bifidobacteria strains: Role of conjugated bile salt hydrolase and pH. Can J Microbiol 46:878–884.

7. Von Ah U, Mozzetti V, Lacroix C, Kheadr EE, Fliss I, Meile L. 2007. Classification of a moderately oxygen-tolerant isolate from baby faeces as *Bifidobacterium thermophilum*. BMC Microbiol 7:1–11.

8. Matsumoto M, Ohishi H, Benno Y. 2004. H^+-ATPase activity in *Bifidobacterium* with special reference to acid tolerance. Int J Food Microbiol 93:109–113.

9. Zhang J, Wang S, Zeng Z, Qin Y, Li P. 2019. The complete genome sequence of *Bifidobacterium animalis* subsp. *lactis* 01 and its integral components of antioxidant defense system. 3 Biotech 9:352.

10. Shen Q, Shang N, Li P. 2011. In vitro and in vivo antioxidant activity of *Bifidobacterium animalis* 01 isolated from centenarians. Curr Microbiol 62:1097–103.

11. Simpson PJ, Stanton C, Fitzgerald GF, Ross RP. 2005. Intrinsic tolerance of *Bifidobacterium* species to heat and oxygen and survival following spray drying and storage. J Appl Microbiol 99:493–501.

12. Oberg TS, Steele JL, Ingham SC, Smeianov V V., Briczinski EP, Abdalla A, Broadbent JR. 2011. Intrinsic and inducible resistance to hydrogen peroxide in *Bifidobacterium* species. J Ind Microbiol

Biotechnol 38:1947–1953.

13. Ruiz L, Gueimonde M, Patricia RM, Ribbera A, de los Reyes-Gavilán CG, Ventura M, Margolles A, Sánchez B. 2012. Molecular clues to understand the aerotolerance phenotype of *Bifidobacterium animalis* subsp. *lactis*. Appl Environ Microbiol 78:644–650.

14. Meile L, Ludwig W, Rueger U, Gut C, Kaufmann P, Dasen G, Wenger S, Teuber M. 1997. *Bifidobacterium lactis* sp. nov., a moderately oxygen tolerant species isolated from fermented milk. Syst Appl Microbiol 20:57–64.

15. Laureys D, Cnockaert M, De Vuyst L, Vandamme P. 2016. *Bifidobacterium aquikefiri* sp. nov., isolated from water kefir. Int J Syst Evol Microbiol 66:1281–1286.

16. Eckel VPL, Ziegler L-M, Vogel RF, Ehrmann M. 2020. *Bifidobacterium tibiigranuli* sp. nov. isolated from homemade water kefir. Int J Syst Evol Microbiol 70:1562–1570.

17. Hayashi K, Maekawa I, Tanaka K, Ijyuin S, Shiwa Y, Suzuki I, Niimura Y, Kawasaki S. 2013. Purification and characterization of oxygen-inducible haem catalase from oxygen-tolerant *Bifidobacterium asteroides*. Microbiology 159:89–95.

18. Foroni E, Duranti S, Serafini F, Viappiani A, Strati F, Ferrarini A, Delledonne M, Henrissat B, Coutinho P, Fitzgerald GF, Margolles A, Sinderen D Van, Ventura M. 2012. *Bifidobacterium asteroides* PRL2011 genome analysis reveals clues for colonization of the insect gut. PLoS One 7:1–14.

19. Kawasaki S, Mimura T, Satoh T, Takeda K, Niimura Y. 2006. Response of the microaerophilic *Bifidobacterium* species, *B. boum* and *B. thermophilum*, to oxygen. Appl Environ Microbiol 72:6854–6858.

20. Killer J, Kopečny J, Mrázek J, Rada V, Benada O, Koppová I, Havlík J, Straka J. 2009. *Bifidobacterium bombi* sp. nov., from the bumblebee digestive tract. Int J Syst Evol Microbiol 59:2020–2024.

21. Scardovi V, Trovatelli LD, Biavati B, Zani G. 1979. *Bifidobacterium cuniculi*, *Bifidobacterium choerinum*, *Bifidobacterium boum*, and *Bifidobacterium pseudocatenulatum*: four new species and their deoxyribonucleic acid homology relationships. Int J Syst Bacteriol 29:291–311.

22. Ventura M, Turroni F, Zomer A, Foroni E, Giubellini V, Bottacini F, Canchaya C, Claesson MJ, He F, Mantzourani M, Mulas L, Ferrarini A, Gao B, Delledonne M, Henrissat B, Coutinho P, Oggioni M, Gupta RS, Zhang Z, Beighton D, Fitzgerald GF, O'Toole PW, Van Sinderen D. 2009. The

Bifidobacterium dentium Bd1 genome sequence reflects its genetic adaptation to the hum an oral cavity. PLoS Genet 5.

23. Modesto M, Michelini S, Stefanini I, Sandri C, Spiezio C, Pisi A, Filippini G, Biavati B, Mattarelli P. 2015. *Bifidobacterium lemurum* sp. nov., from faeces of the ring-tailed lemur (*Lemur catta*). Int J Syst Evol Microbiol 65:1726–1734.

24. Kiviharju K, Leisola M, Von Weymarn N. 2004. Light sensitivity of *Bifidobacterium longum* in bioreactor cultivations. Biotechnol Lett 26:539–542.

25. Xiao M, Xu P, Zhao J, Wang Z, Zuo F, Zhang J, Ren F, Li P, Chen S, Ma H. 2011. Oxidative stress-related responses of *Bifidobacterium longum* subsp. *longum* BBMN68 at the proteomic level after exposure to oxygen. Microbiology 157:1573–1588.

26. Tanaka K, Satoh T, Kitahara J, Uno S, Nomura I, Kano Y, Suzuki T, Niimura Y, Kawasaki S. 2018. O_2-inducible H_2O_2-forming NADPH oxidase is responsible for the hyper O_2 sensitivity of *Bifidobacterium longum* subsp. *infantis*. Sci Rep 8:1–10.
